# Multi‐Omics Analysis Reveals Translational Landscapes and Regulations in Mouse and Human Oocyte Aging

**DOI:** 10.1002/advs.202301538

**Published:** 2023-07-03

**Authors:** Jiana Huang, Peigen Chen, Lei Jia, Tingting Li, Xing Yang, Qiqi Liang, Yanyan Zeng, Jiawen Liu, Taibao Wu, Wenqi Hu, Kehkooi Kee, Haitao Zeng, Xiaoyan Liang, Chuanchuan Zhou

**Affiliations:** ^1^ Reproductive Medicine Center The Sixth Affiliated Hospital of Sun Yat‐sen University Guangzhou 510655 China; ^2^ Guangdong Engineering Technology Research Center of Fertility Preservation Guangzhou 510610 China; ^3^ Biomedical Innovation Center The Sixth Affiliated Hospital, Sun Yat‐sen University Guangzhou 510655 China; ^4^ Center for Stem Cell Biology and Regenerative Medicine, Department of Basic Medical Sciences, School of Medicine Tsinghua University Beijing 100084 China

**Keywords:** Hells, m6A modifications, oocyte aging, translatomics, YTHDF3

## Abstract

Abnormal resumption of meiosis and decreased oocyte quality are hallmarks of maternal aging. Transcriptional silencing makes translational control an urgent task during meiosis resumption in maternal aging. However, insights into aging‐related translational characteristics and underlying mechanisms are limited. Here, using multi‐omics analysis of oocytes, it is found that translatomics during aging is related to changes in the proteome and reveals decreased translational efficiency with aging phenotypes in mouse oocytes. Translational efficiency decrease is associated with the N6‐methyladenosine (m6A) modification of transcripts. It is further clarified that m6A reader YTHDF3 is significantly decreased in aged oocytes, inhibiting oocyte meiotic maturation. YTHDF3 intervention perturbs the translatome of oocytes and suppress the translational efficiency of aging‐associated maternal factors, such as *Hells*, to affect the oocyte maturation. Moreover, the translational landscape is profiled in human oocyte aging, and the similar translational changes of epigenetic modifications regulators between human and mice oocyte aging are observed. In particular, due to the translational silence of YTHDF3 in human oocytes, translation activity is not associated with m6A modification, but alternative splicing factor SRSF6. Together, the findings profile the specific translational landscapes during oocyte aging in mice and humans, and uncover non‐conservative regulators on translation control in meiosis resumption and maternal aging.

## Graphical abstract

The study analyzes transcriptomes, translatomes, and proteomes of mouse and human GV oocytes at the single‐cell level with a focus on conservations and differences in maternal aging between the two species. N6‐methyladenosine (m6A) reader YTHDF3 and alternative splicing factor SRSF6 are associated with the aging‐associated translational control in meiosis resumption in mice and humans, respectively.

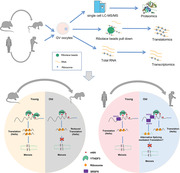

## 1 Introduction

Female reproductive aging has become an urgent public health concern with women globally delaying childbearing, which is linked to infertility, obstetric complications, and perinatal risks.^[^ ^1^ ^]^ The chief causes of female age‐related infertility include decreased ovarian reserve, abnormal initiation of meiotic resumption, diminished oocyte quality, and reduced oocyte/embryo competence.^[^ ^2^, ^3^, ^4^ ^]^ In particular, compared to the diminished oocyte reserve due to ovary aging, the decline in oocyte quality with abnormal initiation of meiotic resumption, oocyte meiotic maturation errors, and aberrant oocyte‐to‐embryo transition (OET) competence play a more significant role in aged‐related infertility,^[^ ^5^, ^6^ ^]^ which makes oocyte aging a nonnegligible issue in female reproductive aging. Various exogenous and endogenous factors contribute to aging‐related oocyte quality and competence issues, including DNA repair apparatus dysfunction, impaired spindle assembly checkpoint integrity, cohesion ring loss, chromosomal abnormalities, mitochondrial damage, and oxidative stress.^[^ ^7^, ^8^, ^9^, ^10^, ^11^, ^12^ ^]^ The aberrant establishment and accumulation of epigenetic marks on DNA and chromatin during oocyte aging is an additional aspect affecting oocyte quality and competence; this is called epigenetic maturation dysfunction^[^ ^13^ ^]^ and has effects such as reduced trimethylation of histone H3 at lysine‐4 (H3K4me3), increased acetylation on lysine 12 of histone H4,^[^ ^6^, ^14^, ^15^ ^]^ and aberrant DNA methylation.^[^ ^13^, ^16^, ^17^, ^18^ ^]^ Whereas some of the basic mechanisms of oocyte aging have been elucidated, little is known about the complicated regulated processing of maternal mRNAs in mammalian oocytes during aging, including humans.

The development of single‐cell transcriptome methods has been widely used in oocyte aging studies. Single‐cell RNA sequencing (RNA‐seq) studies in aged human and mouse oocytes revealed alterations in transcripts involved in the cell cycle, RNA splicing, and RNA decay.^[^ ^19^, ^20^, ^21^ ^]^ However, in mammalian oocyte development, primordial oocytes periodically grow rapidly along with accumulating abundant mRNAs and proteins. Unlike somatic cells, fully grown oocytes at the germinal vesicle (GV) stage undergo transcriptional cessation upon meiosis resumption,^[^ ^22^, ^23^ ^]^ and the orchestrated regulation of posttranscriptional modification and RNA translation is essential for meiotic maturation and OET. In previous studies, this has usually produced inconsistencies between transcriptomics and proteomics. For example, increased *sirt1* transcription levels and reduced protein levels have been observed in aged mouse oocytes.^[^ ^24^ ^]^ Reduced DNA methylation in aged mouse oocytes has been widely reported, whereas the corresponding transcription levels remain constant.^[^ ^18^ ^]^ These phenomena indicate the limits of utilizing transcriptomics to investigate oocyte aging. On the other hand, although mass spectrometry enables the detection of the protein expression of functional genes, limitations still exist because only proteins that number in the thousands can be identified, and low‐abundance critical factors might therefore be missed.^[^ ^25^, ^26^ ^]^

Recently, great advances have been made in single‐cell techniques that can satisfy translational investigation needs by using a few oocytes/embryos. For instance, Zhang et al.^[^ ^27^ ^]^ reported a low‐input LiRibo‐seq that can enable the exploration of translational dynamics with 100–250 oocytes/embryos. Furthermore, an ultrasensitive Ribo‐seq technique using 30–150 oocytes/embryos was reported by Xiong et al.^[^ ^22^ ^]^ A recent study with 200 aged mouse oocytes demonstrated the translational dynamics alterations involved in meiotic spindle assembly and chromosome segregation.^[^ ^28^ ^]^ However, due to the restrictions of cell abundance requirements, these methods are not feasible for human oocyte studies. Numerous studies have elucidated the connections between aging and ribosome dysregulation.^[^ ^29^, ^30^ ^]^ Aging exacerbates ribosome pausing and alters translation efficiency to impair proteostasis.^[^ ^31^ ^]^ Additionally, the translation elongation rates decline with age.^[^ ^32^ ^]^ Nevertheless, the translational landscapes and regulations in mammalian oocytes during aging, especially human oocytes, have not been reported. The prevalent trend of globally delaying childbearing in human females and the impacts of aging on oocyte quality presents a critical demand to elucidate its underlying mechanisms and provide a novel interventional strategy. Notably, we have developed a novel single‐cell T&T‐seq method that can be used to conduct transcriptome and translatome analyses using 1 to 10 oocytes.^[^ ^25^ ^]^ This method enables us to portray the translational and transcriptional landscapes in mammalian oocyte aging with single oocytes. The precise posttranscriptional modifications and the translations of prestored mRNA are vital assurances for oocyte meiotic maturation, OET and early embryo development.^[^ ^33^, ^34^, ^35^, ^36^ ^]^ N6‐methyladenosine (m6A) is the most abundant modification of mRNA in eukaryotes^[^ ^37^, ^38^ ^]^ and is reversibly regulated by m6A writers (methyltransferases, which deposit), erasers (demethylases, which remove), and readers (which recognize).^[^ ^39^ ^]^ In recent years, emerging studies have addressed the crucial role m6A writers, readers, and erasers play in oocyte developmental competence by regulating mRNA alternative splicing,^[^ ^40^ ^]^ stability,^[^ ^23^ ^]^ degradation,^[^ ^41^ ^]^ and polyadenylation^[^ ^42^ ^]^ in oocytes. M6A is critical in the regulation of RNA stability in oocytes and in timely RNA degradation during the maternal to zygotic transition (MZT).^[^ ^43^ ^]^ A recent investigation also revealed that m6A modulates the chromatin state and gene expression during oocyte/embryonic development by regulating retrotransposon RNA transcription and degradation.^[^ ^44^, ^45^, ^46^ ^]^ Importantly, it has been elucidated that m6A facilitates RNA translation via its reader YTH N6‐methyladenosine RNA‐binding protein 3 (YTHDF3) in synergy with YTHDF1.^[^ ^37^ ^]^ In addition, studies have revealed that senescent human mesenchymal stem cell models and mouse models of Alzheimer's disease exhibit decreased m6A methylation levels.^[^ ^47^, ^48^ ^]^ However, whether m6A mediates RNA translation in oocytes and what the functions of m6A are in oocyte aging remain largely unknown.

In this study, we aimed to investigate the precise translational landscapes and the functions of translational regulatory factors during oocyte aging by analyzing the single‐cell translatome and transcriptome in GV‐stage oocytes from young and aged female mice/humans. Additionally, the conservations and differences of oocyte aging between two species are investigated via integrated analysis of the translatomics and translational regulators alterations during oocyte aging from two species, which may help to provide specific intervention strategies of oocyte aging to two species. Here, we demonstrated the landscapes of mouse oocyte aging by conducting multi‐omics analysis on oocytes, including single‐cell proteomics, translatomics and transcriptomics. Further, we investigated the role of m6A reader YTHDF3 in mouse oocyte aging and identified a crucial m6A‐containing YTHDF3‐binding target *Hells*. Moreover, the translational landscapes of human aging oocytes were also explored by using single‐cell translatome and transcriptome analysis. The cross‐species conservations and differences between aged mouse and human oocytes in RNA translation were identified.

## 2 Results

### 2.1 Multi‐Omics Analysis of Aged and Young Mouse Oocytes

**Figure 1:**
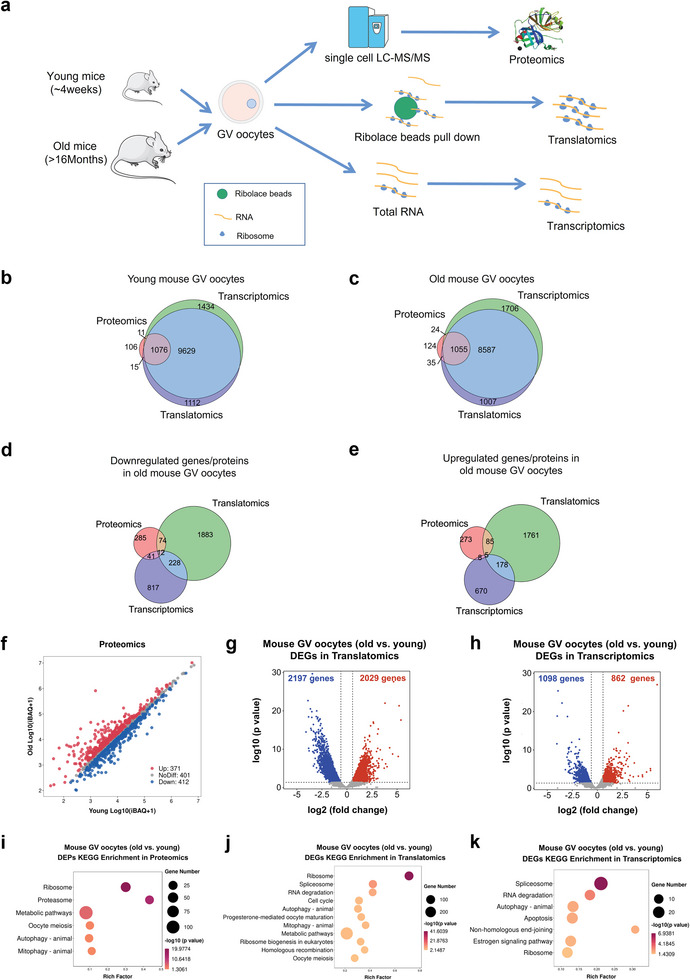
Single‐cell proteomics, ultrasensitive translatomics, and transcriptomics of aged and young mouse oocytes. a) Schematic graph depicting the major procedures of multi‐omics profiling in aged and young mouse GV oocytes. For single‐cell proteome profiling, one biological replicate of 1 oocyte was performed; for ultrasensitive transcriptomics and translatomics profiling, three biological replicates of 4 oocytes were generated. b,c) Venn plot of detected genes generated by single‐cell translatomics (TPM>1 for any biological replicate), RNA‐seq (TPM>1 for any biological replicate), and proteome‐identified genes in young mouse oocytes (≈4 weeks old) and in aged mouse oocytes (>16 months old). d) Venn diagram showing the overlap of genes downregulated in aged mouse GV oocytes detected from the ultrasensitive translatomics (FC<0.67), RNA‐seq (FC<0.67), and proteome (FC<0.83). e) Venn diagram showing the overlap of genes upregulated in aged mouse GV oocytes detected from the ultrasensitive translatomics (FC>1.5), RNA‐seq (FC>1.5), and proteome (FC>1.2). f) Scatter plot demonstrating the differentially expressed proteins between aged and young mouse GV oocytes. Red and blue dots denote up‐ and down‐regulated proteins, respectively. g,h) Volcano diagram showing DEGs detected by ultrasensitive translatomics (g) and transcriptomics (h). Red and blue dots denote up‐ and down‐regulated genes, respectively. *p* < 0.05, FC>1.5 or <0.67. i–k) Representative KEGG analysis of the differentially expressed proteins/DEGs detected by proteomics (i), translatomics (j), and RNA‐seq (k). TPM, transcripts per million. DEGs, differentially expressed genes. KEGG, Kyoto Encyclopedia of Genes and Genomes.

The predominant causes of female age‐related fertility decline are reduced oocyte quantity and quality. In this study, we observed that the oocyte retrieval quantity and in vitro polar body‐1 (PB1) emission rates were significantly decreased in aging mice (>16 months old) compared with young mice (≈4 weeks old; Figure S1a,b, Supporting Information). To better understand the underlying mechanisms that cause the decreased oocyte quality/competence of aged oocytes, we applied single‐cell proteome profiling, ultrasensitive translatomics, and total RNA‐seq to GV‐stage oocytes from young and old female mice (**Figure** **1**a). For single‐cell proteome profiling, one biological replicate was performed. For ultrasensitive translatomics and RNA‐seq, we generated three biological replicates (4 oocytes for one translatome and transcriptome), which presented high reproducibility (Figure S1c,d, Supporting Information). Additionally, principal component analysis (PCA) revealed distinct clusters between young and aging mouse oocytes, either in translatomics (Figure S1e, Supporting Information) or transcriptomics (Figure S1f, Supporting Information). In total, we detected ≈10 741 genes [transcripts per million (TPM) >1] and 9594 genes (TPM>1) in young and aging mouse oocytes, respectively, that actively engaged in translation; these quantities were approximately 10‐fold higher than the number of proteins detected by single‐cell proteomics analysis. More importantly, over 90% of the young mouse GV proteins and 88% of the aging mouse GV proteins were detected by translatomics profiling (Figure 1b,c), indicating a higher sensitivity and coverage of translatomics in oocyte research. Moreover, we compared and identified overlap in the downregulated or upregulated proteins/genes identified by single‐cell proteomics, translatomics, and transcriptomics. Compared with transcriptomics, the alteration trends of gene expression detected by translatomics analysis were more consistent with proteomics in aged mouse oocytes (Figure 1d,e; Figure S1g, Supporting Information). These findings suggest that the translatome is a better tool than transcriptome for investigating protein expression in oocytes during maternal aging. In addition, we carried out ultrasensitive translatomics on young and aged GV oocytes and MII oocytes. The translatomics of representative genes, which regulate maternal transcriptome and mRNA translational activation during oocyte meiotic maturation, obtained in our study were compared with the protein expression levels reported in previous literature.^[^ ^35^, ^36^, ^49^, ^50^ ^]^ Consistent with previous studies, the expression levels of *Btg4*, *Cnot7*, *Cnot8*, and *Ccnb1* were upregulated during oocyte meiotic maturation, while the expression of *Zar1l* and *Cpeb1* were downregulated (Figure S1h, Supporting Information). These findings confirmed that the ultrasensitive translatomics in this study were valuable to reflect proteomics in mammalian oocytes. Next, scatter plots and volcano plots were generated to present the activated and repressed proteins/genes identified by proteomics, translatomics, and transcriptomics in the aged mouse oocytes (Figure 1f–h). Intriguingly, compared with transcriptomics, in translatomics the variations between aged oocytes and young oocytes were much greater, which is consistent with the classic theory of transcriptional silencing during mammalian oocyte meiotic maturation. Kyoto Encyclopedia of Genes and Genomes (KEGG) analysis showed that, in aged mouse oocytes, the “ribosome” and “autophagy” pathways were consistently enriched in these differentially expressed proteins (DEPs)/genes (DEGs). However, notably, translatomics analysis provided more abundant pathway enrichment information and indicated that translational dysregulation in aged oocytes was associated with “progesterone‐mediated oocyte maturation,” “homologous recombination,” and “oocyte meiosis” (Figure 1i–k).

### 2.2 Translational Dynamics in Aged Mouse Oocytes

**Figure 2:**
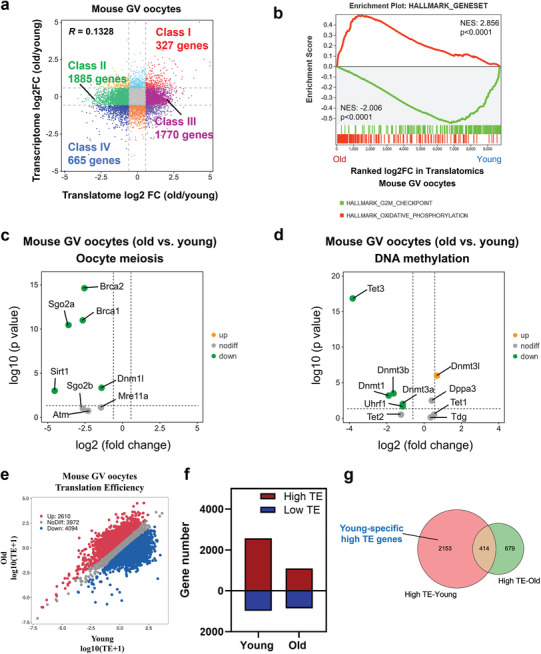
Distinct translational patterns in aged and young mouse GV oocytes. a) Scatter plot showing the changes in gene translation and transcription during oocyte aging. Class I (red) denotes genes with upregulated translation and transcription in aged mouse GV oocytes. Class II (green) denotes genes translationally downregulated in aged mouse GV oocytes but transcriptionally constant. Class III (purple) denotes genes translationally upregulated in aged mouse GV oocytes but transcriptionally constant. Class IV (blue) denotes genes with downregulated translation and transcription in aged mouse GV oocytes. Downregulated, FC<0.67; upregulated, FC>1.5. b) Gene set enrichment analysis of the translatomics showing the translationally downregulated genes enriched in the hallmark of the G2/M checkpoint and translationally upregulated genes enriched in the hallmark of oxidative phosphorylation. c) Volcano plot showing the changes in oocyte meiosis‐related genes identified by the translatomics. d) Volcano plot showing the changes in the DNA methylation regulatory genes identified by the translatomics. e) Scatter plot showing the RNA TE alterations of aged mouse oocytes compared with young mouse oocytes. Red and blue dots denote up‐ and down‐regulated genes, respectively. Upregulated, FC>1.5; downregulated, FC<0.67. f) Bar plots showing the numbers of high‐TE genes (TE>2) and low‐TE genes (TE<0.5) in young and aged mouse GV oocytes, respectively. g) Venn diagram showing the overlap of high‐TE genes identified from young and aged mouse GV oocytes. TE, translation efficiency. FC, fold change.

To better characterize the translational and transcriptional dynamics in oocytes during maternal aging, we next performed a combined analysis with the gene expression data (TPM>1) in aged and young oocytes detected by translatomics and transcriptomics (**Figure** **2**a). Notably, the *Pearson* correlation coefficient between gene translation and transcription was only 0.1328. Most aged oocyte‐specific translationally repressed (class II, 1885 genes) or enriched (class III, 1770 genes) genes had constant transcriptional expressions, which is consistent with the conventional theory of transcriptional inactivation in fully grown oocytes.

Furthermore, we applied gene set enrichment analysis (GSEA) to these differentially translated genes in aged oocytes. Specifically, GSEA demonstrated that, in aged mouse oocytes, the oxidative phosphorylation pathway was enriched in the actively translated genes, while the G2/M checkpoint process was enriched in the translationally repressed genes, suggesting that the increased oxidative stress and meiotic arrest in aged oocytes may be attributable to translational dysregulation (Figure 2b). Mitochondrial damage and DNA repair apparatus dysfunction play critical roles in age‐related oocyte meiotic errors.^[^ ^7^, ^51^, ^52^ ^]^ Hence, we further analyzed the expression of meiotic regulatory factors. Intriguingly, the translational levels of represented meiosis‐related genes, including *Brca1*, *Brca2*, *Sgo2a*, *Sirt1*, and *Dnmt1*, in aged mouse oocytes decreased significantly, while the transcription levels remained unchanged (Figure 2c; Table S1, Supporting Information). These findings support the notion that the downregulated translation of specific genes accounts for mitochondrial damage and DNA repair apparatus dysfunction in aged oocytes.

More importantly, timely RNA decay and epigenetic transitions, such as histone modifications and DNA methylations, are essential for oocyte meiotic resumption and maturation, MZT, and zygotic genome activation (ZGA).^[^ ^53^, ^54^, ^55^ ^]^ Additional translations of undegraded mRNAs lead to microtubule‐chromosome organization errors and meiotic cell cycle arrest.^[^ ^56^ ^]^ We observed that the translational levels of key maternal mRNA degradome regulators, such as *Cnot6 l/6/8*, *TUT4/7*, and *Btg4*, decreased significantly in aged mouse oocytes (Figure S2a, Supporting Information). However, inconsistent with a previous report,^[^ ^6^ ^]^ the transcription levels of these genes in aged oocytes remained unchanged (Table S1, Supporting Information). Moreover, consistent with previous reports that the DNA methylation levels decreased during aging,^[^ ^17^, ^18^ ^]^ the translational levels of key regulators of DNA methylation, such as *Dnmt3a/3b*, *Dnmt1*, and *Uhrf1*, were drastically reduced in aged oocytes (Figure 2d). Notably, the establishment of DNA methylation and regulation of RNA degradation was largely associated with histone modifications. For instance, histone 3 lysine 4 (H3K4) demethylation by KDM1A or KDM1B is essential for timely methylation establishment at CpG islands.^[^ ^57^ ^]^ In addition, timely mRNA clearance partly depends on a network of CxxC‐finger protein 1‐maintained H3K4me3.^[^ ^6^ ^]^ The deficiency of H3K4 demethylases (KDM1A or KDM1B) results in oocyte meiotic resumption/progression errors and MZT and ZGA defects.^[^ ^58^, ^59^, ^60^ ^]^ In accordance with these findings, we observed dramatic translational repression of *Kdm1a/b* in aged mouse oocytes (Figure S2b, Supporting Information). The methylase EZH2 of histone H3 Lys 27 trimethylates (H3K27me3) is essential for mouse oocyte meiotic maturation, and its depletion causes chromosome aneuploidy.^[^ ^61^ ^]^ In this study, the translational levels of *Ezh2* were also reduced in aging mouse oocytes (Figure S2b, Supporting Information). Interestingly, we observed that crucial mediators of histone crotonylation, including *Crebbp*, *Cyd1*, and *Ep300*, were generally downregulated (Figure S2b, Supporting Information). Noticeably, the transcription of key factors of histone modifications also remained unchanged (Table S1, Supporting Information). Although the functions of histone crotonylation in oocytes have not been elucidated, these results suggest that histone crotonylation may participate in decreased oocyte quality/competence during aging, which warrants further investigation. Overall, the study illustrates the decreased translations of transcripts encoding key regulators of RNA decay, DNA methylation, and histone modifications, which helps to explain the underlying mechanisms of decreased quality/competence in mouse oocytes during aging. More importantly, we also provide novel insight into the role of histone crotonylation in oocyte aging, which requires further investigation.

### 2.3 Translational Efficiency (TE) Alterations in Aged Mouse Oocytes

As the correlation between translatomics and transcriptomics was low, we further analyzed the TE of highly expressed genes (TPM>1 in transcriptomics) in aged and young mouse oocytes. We found that the translation efficiency of a large number of genes was inhibited, with 4094 and 2610 genes down‐ and upregulated in aged mouse oocytes, respectively (Figure 2e). Consistent with the DEGs in translatomics, GSEA demonstrated that in aged mouse oocytes, the oxidative phosphorylation pathway was enriched in the genes with elevated TE, while the G2/M checkpoint process was enriched with the genes with reduced TE (Figure S2c, Supporting Information). Interestingly, 2567 young oocyte GV‐enriched genes had a TE>2 (high‐TE), while only 1093 aged oocyte GV‐enriched genes had a TE>2, showing that the numbers of high‐TE genes were drastically decreased in aged mouse oocytes. On the other hand, the number of genes with TE<0.5 (low‐TE) in young oocytes (1093 genes) was similar to that in aged oocytes (855 genes) (Figure 2f), indicating that TE inhibition in aged mouse oocytes generally occurs in genes with high‐TE. Next, we compared the high‐TE genes in aged and young mouse oocytes and found that only 414 genes overlapped (Figure 2g). Notably, gene ontology (GO) analysis indicated that young oocyte‐specific high‐TE genes were associated with “metabolic process,” “chromosome organization” and “cell cycle” (Figure S2d, Supporting Information), which suggests that the repression of high‐TE genes may be related to higher rates of meiotic resumption failure and aneuploidy in aged mouse oocytes.

### 2.4 The Role of M6A Modification in Regulating TE

**Figure 3:**
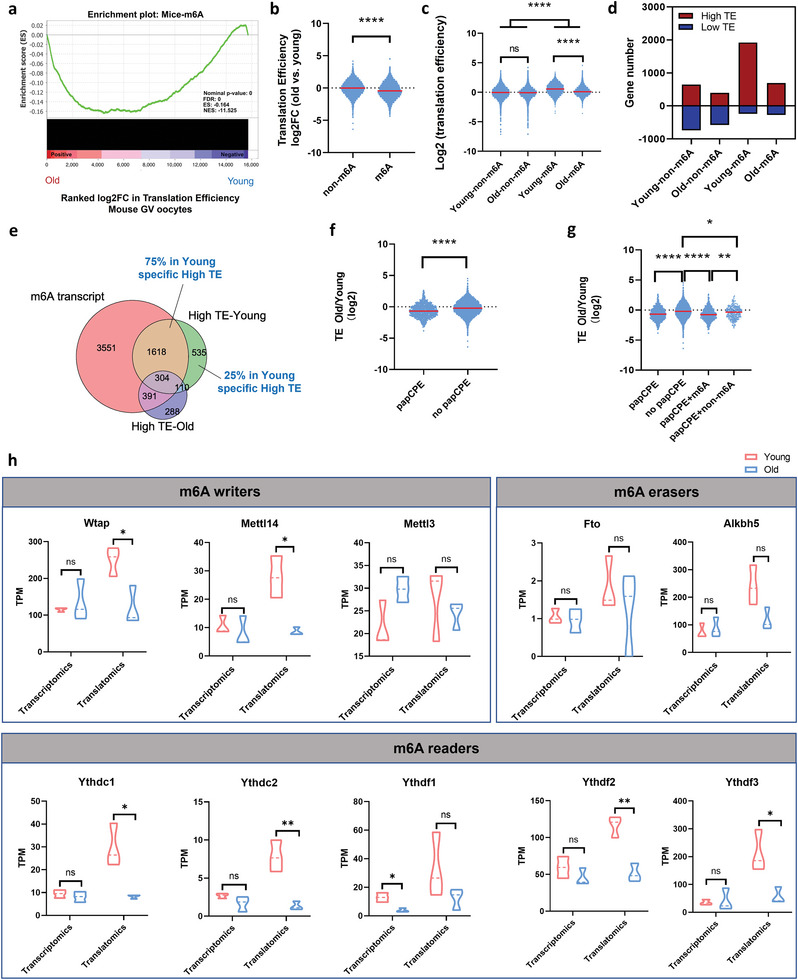
M6A modification that mediates translation downregulation is altered in aged mouse GV oocytes. a) Gene set enrichment analysis showing that the TE of m6A‐enriched RNA was significantly decreased. b) Violin plots showing the TE changes in the genes of the group not enriched by m6A and of the m6A‐enriched group in aged mouse oocytes compared with young mouse oocytes. *p*‐Value was calculated with Student's t‐test for independent samples. c) Violin plots showing TE (log2) in young and aged mouse oocytes for m6A‐enriched genes or in genes not enriched by m6A. *p*‐Values were calculated with one‐way ANOVA and Bonferroni post hoc test. d) Bar plots showing the numbers of high‐TE genes and low‐TE genes among the four groups. The minus sign indicates the number of downregulated genes. e) Venn diagram showing the overlap of the m6A‐enriched gene set, high‐TE genes in young mouse GV oocytes, and high‐TE genes in aged mouse GV oocytes. f) Violin plots showing the TE changes in genes of the papCPE‐containing group and in genes of the group not containing papCPE in aged mouse oocytes compared with young mouse oocytes. *p*‐Value was calculated with Student's t‐test for independent samples. g) Violin plots showing the TE changes in four groups of genes in aged mouse oocytes compared with young mouse oocytes. *p*‐Values were calculated with one‐way ANOVA and Bonferroni post hoc test. h) Transcriptional and translational expression levels of the representative m6A‐related genes in mouse GV oocytes. Data are shown as the mean ± SEMs. *p*‐Values were calculated with Student's t‐test for independent samples. TE, translational efficiency. papCPE, cytoplasmic polyadenylation elements (CPEs) within 100 bp of PASs. Ns, no significant difference. *p < 0.05, **p < 0.01, ****p < 0.0001.

We then sought to understand how TE was translationally downregulated in aged mouse oocytes. M6A, the most prevalent RNA modification in eukaryotes, plays a key role in regulating RNA translation.^[^ ^37^, ^62^, ^63^ ^]^ Indeed, we found that the TE of m6A‐enriched RNA was globally decreased in aged mouse oocytes (**Figure** **3**a,b). In addition, when comparing the TE in young oocytes with that in aged oocytes, only the TE of m6A‐modified RNA showed significant differences between young and aged oocytes, and aged oocytes held lower TE, while the TE of RNA not modified by m6A were not significantly changed (Figure 3c, Figure S3a, Supporting Information). More importantly, the abundance of high‐TE m6A‐modified genes was drastically decreased in aged mouse oocytes, while the numbers of low‐TE m6A‐enriched genes and high/low‐TE genes not modified by m6A were similar in aged and young oocytes (Figure 3d). In addition, 75% of young oocyte‐specific high‐TE transcripts contained m6A modification (Figure 3e). Therefore, these analyses reveal a vital role of m6A modification in translation regulation in mouse oocytes during aging.

In particular, the presence of cytoplasmic polyadenylation elements (CPEs) and polyadenylation signals (PASs) is crucial for translational regulation. CPE and papCPE (CPEs within 100 bp of PASs) have accumulative translationally repressive effects in fully grown oocytes.^[^ ^22^ ^]^ In this study, the analysis of CPE and TE changes during oocyte aging revealed no or low correlation (Figure S3b, Supporting Information). Correspondingly, TE was significantly repressed in transcripts with papCPE (Figure 3f). Interestingly, we found that most of the papCPE‐containing genes were m6A‐modified. The repressive effects of papCPE were correlated with m6A modification (Figure 3g). Such repressive effects were much weaker for RNA not enriched by m6A (Figure 3g). Hence, these findings also highlighted the critical role of m6A in translational regulation.

Next, the transcriptional and translational expression levels of m6A‐associated genes in aged and young mouse oocytes were evaluated. Notably, the translational levels of most m6A writers and readers, including *Wtap*, *Mettl14*, *Ythdc1*, *Ythdc2*, *Ythdf2*, and *Ythdf3*, were dramatically decreased in aged mouse oocytes, while their transcription remained constant (Figure 3h). In particular, the basic translational levels of *Wtap*, *Ythdf2*, and *Ythdf3* were most abundant (Figure 3h). Overall, in aged mouse oocytes, m6A modifications play a key role in RNA TE downregulation.

### 2.5 YTHDF3 Depletion Suppressed RNA TE in Mouse Oocytes

**Figure 4:**
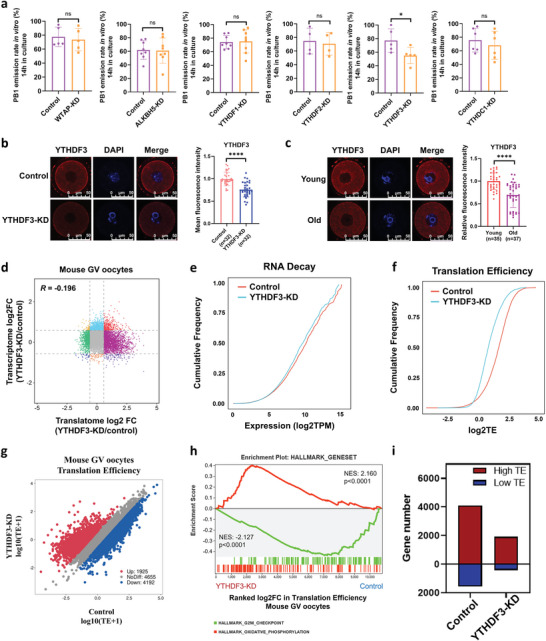
YTHDF3 deficiency inhibits RNA TE in mouse oocytes. a) The in vitro PB1 emission rates of mouse oocytes from the control groups and the m6A‐related gene depletion groups. Each dot represents a single biological replicate. *p*‐Values were calculated with Student's t‐test for paired samples. b) Immunofluorescence verifying the depletion of YTHDF3 by Trim‐Away. Scale bar, 50 µm. The right panel shows the quantification of YTHDF3 protein levels. The average intensity of the control group oocytes was set as 1.0. Each dot represents a single oocyte analyzed. *p*‐Value was calculated with two‐tailed Mann–Whitney test. c) Immunofluorescence verifying the expression of YTHDF3 in young and aged mouse GV oocytes. Scale bar, 50 µm. The right panel shows the quantification of YTHDF3 protein levels. The average intensity of young mouse oocytes was set as 1.0. Each dot represents a single oocyte analyzed. *p*‐Value was calculated with two‐tailed Mann–Whitney test. d) Scatter plot showing the changes in gene translation and transcription in YTHDF3‐KD oocytes and control oocytes. The Pearson correlation coefficient = −0.196. e) Cumulative distribution of total RNA expression (log_2_ TPM); the red line denotes the control group, and the blue line represents the YTHDF3‐KD group. f) Cumulative distribution of TE. The red line denotes the control group, and the blue line represents the YTHDF3‐KD group. g) Scatter plot showing the RNA TE alterations of YTHDF3‐KD oocytes compared with the control group. Red and blue dots denote up‐ and down‐regulated genes, respectively. Upregulated, FC>1.5; downregulated, FC<0.67. h) Gene set enrichment analysis of TE showing the TE downregulated genes enriched in the hallmark of the G2/M checkpoint and TE upregulated genes enriched in the hallmark of oxidative phosphorylation. i) Bar plots showing the numbers of high‐TE genes (TE>2) and low‐TE genes (TE<0.5) in the YTHDF3‐KD group and the control group oocytes, respectively. PB1, polar body‐1. TE, translational efficiency. FC, fold change. YTHDF3‐KD, YTHDF3 knockdown. Ns, no significant difference. *p < 0.05, ****p < 0.0001.

To assess the function of m6A‐associated genes in oocyte quality and developmental competence, we next performed electroporation to degrade target proteins in oocytes using the Trim‐away method and assessed the in vitro PB1 emission rates.^[^ ^64^ ^]^ Surprisingly, only when YTHDF3 was knocked down (YTHDF3 KD) were the in vitro PB1 emission rates of oocytes significantly reduced (**Figure** **4**a). Immunofluorescence verified that the expression of YTHDF3 was dramatically downregulated in YTHDF3‐deficient oocytes (Figure 4b). We also confirmed the significant downregulation of YTHDF3 in aged mouse oocytes by immunofluorescence (Figure 4c). Additionally, immunofluorescence showed the protein expression of YTHDF3 remained stable during oocyte maturation (Figure S4, Supporting Information). These results reveal that the m6A reader YTHDF3 is functional in oocyte quality and developmental competence in mice.

To explore whether YTHDF3 regulates mouse oocyte quality/competence by mediating RNA TE, we next performed ultrasensitive translatomics and transcriptomics (three biological replicates) on oocytes from the YTHDF3‐KD and control groups, which exhibited high reproducibility and distinct clusters (Figure S5a–d, Supporting Information). Similar to the above findings, the gene expression in the translatomics was hardly correlated with that in the transcriptomics, with a *Pearson* correlation coefficient of −0.196 (Figure 4d). As the volcano plots showed, YTHDF3 depletion resulted in 233 genes being downregulated and 993 genes upregulated in the translatomics (Figure S5e, Supporting Information), while 194 genes were downregulated and 125 genes upregulated in the transcriptomics (Figure S5f, Supporting Information). In translatomics analysis, KEGG annotations for the DEGs included “ribosome,” “mitophagy,” “metabolic pathways,” “mismatch repair,” “DNA replication,” and “spliceosome,” whereas in transcriptomics analysis, the KEGG annotations for the DEGs included “metabolic pathways,” “RNA degradation,” “estrogen signaling pathway,” and “oocyte meiosis” (Figure S5g,h, Supporting Information). In addition, consistent with the aged mouse oocytes (Figure 2b), GSEA demonstrated that the actively translated genes detected by translatomics were associated with “oxidative phosphorylation,” while the repressively translated genes were correlated with the “G2/M checkpoint” (Figure S5i, Supporting Information).

Interestingly, by analyzing the genome‐wide changes in RNA expression and TE, we found that YTHDF3 depletion slightly reduced RNA decay in oocytes (Figure 4e). More strikingly, the cumulative frequency curve of RNA TE showed a more obvious change, indicating that YTHDF3 depletion led to a dramatically decreased TE in oocytes (Figure 4f). Hence, we further analyzed the TE in oocytes after YTHDF3 KD. Notably, similar to the RNA TE in aged mouse oocytes (Figure 2e), the TE of most RNA in YTHDF3‐KD was downregulated (4192 genes), above twofold of upregulated TE RNA (1925 genes) (Figure 4g). GSEA indicated that “oxidative phosphorylation” was enriched in the upregulated TE genes, whereas “G2/M checkpoint” was enriched in the downregulated TE genes (Figure 4h). More importantly, compared with the control group (4108 genes), YTHDF3 KD oocytes (1914 genes) had a sharply decreased number of high‐TE genes (Figure 4i). In contrast, the low‐TE genes were also reduced in YTHDF3 KD oocytes (422) compared with the control group (1559) (Figure 4i). In addition, 92% of high‐TE genes in YTHDF3‐KD oocytes overlapped with high‐TE genes in the control group (Figure S5j, Supporting Information).

Collectively, these findings indicate a crucial role of YTHDF3 in regulating RNA TE and oocyte quality/meiotic resumption. The deficiency of YTHDF3 in aged mouse oocytes may account for their age‐related diminished oocyte quality and TE inhibition.

### 2.6 YTHDF3 Modulates RNA TE in an M6A‐Dependent Manner

**Figure 5:**
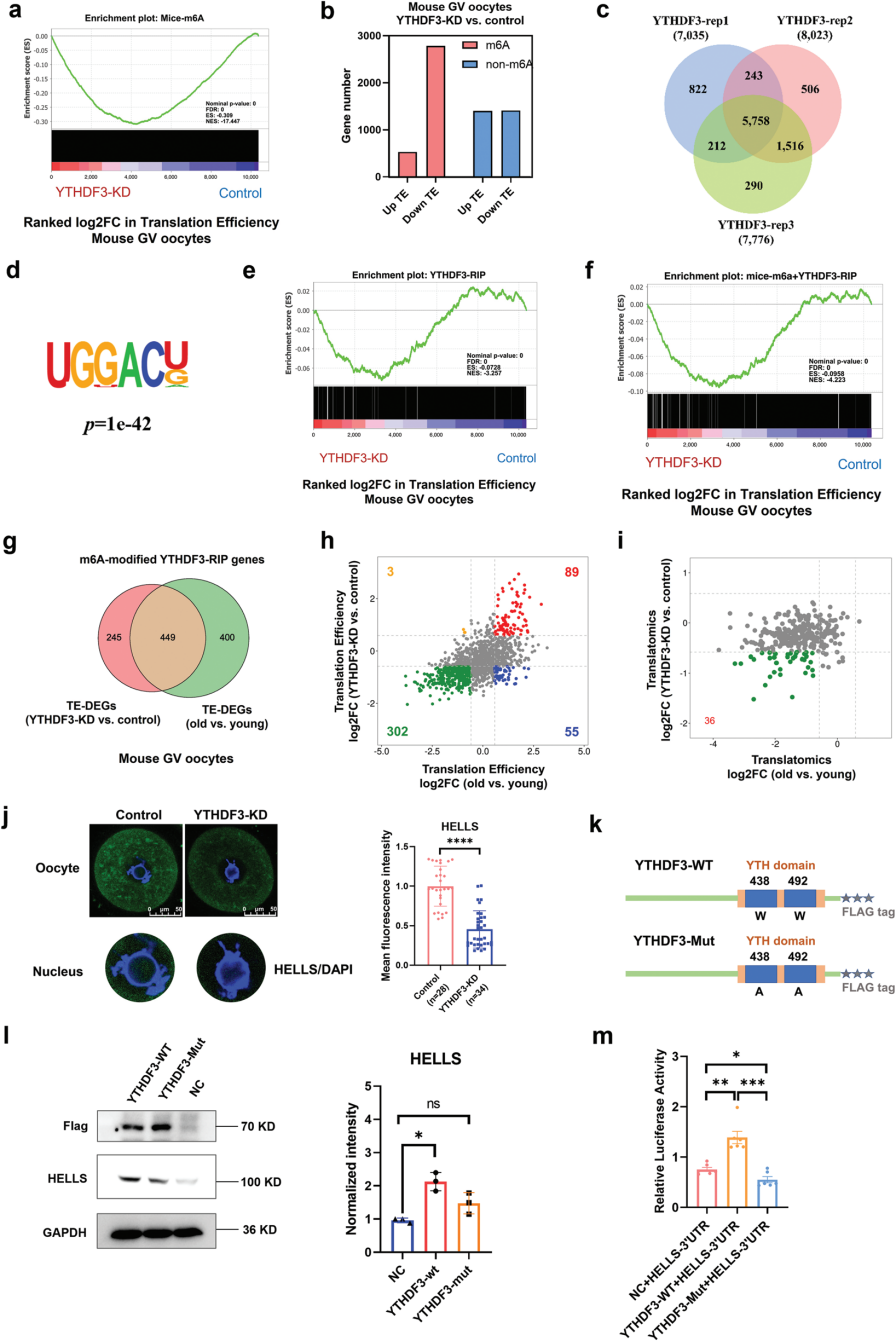
YTHDF3 modulates RNA translation efficiency in an m6A‐dependent manner. a) Gene set enrichment analysis demonstrating that the TE of m6A‐enriched RNA was significantly decreased upon YTHDF3 depletion. b) Bar plots showing the numbers of up‐ (FC>1.5) and down‐regulated (FC<0.67) genes for m6A‐enriched genes or genes not enriched by m6A, respectively. Pink denotes m6A‐enriched genes. Blue denotes genes not enriched by m6A. c) Venn diagram portraying the overlap of YTHDF3 target genes among three independent RIP‐seq biological replicates. d) Motif identified by HOMER within YTHDF3 RIP‐seq peaks in HEK293T cells. e) Gene set enrichment analysis showing the TE alterations of YTHDF3‐binding RNA upon YTHDF3 depletion. f) Gene set enrichment analysis showing the TE alterations of m6A‐enriched YTHDF3‐binding RNA upon YTHDF3 depletion. g) Venn diagram showing the overlap of m6A‐modified YTHDF3 target genes between the differential TE genes in YTHDF3‐KD oocytes and the differential TE genes in aged mouse oocytes. h) The RNA TE log_2_fold change in the 449 overlapping genes (described in g) in aged mouse oocytes and YTHDF3‐depleted oocytes. i) The RNA translational level changes in 302 downregulated TE genes (described in h) in aged mouse oocytes and YTHDF3‐depleted oocytes. j) Immunofluorescence verifying the expression of HELLS in the control group and the YTHDF3‐depleted group oocytes. Scale bar, 50 µm. A screenshot of the nucleus is shown separately at the bottom. The right panel shows the quantification of the HELLS protein level. The average intensity of the control group oocytes was set as 1.0. Each dot represents a single oocyte analyzed. *p*‐Value was calculated with two‐tailed Mann‐Whitney test. k) Schematic representation of wild‐type (YTHDF3‐WT) and mutant (YTHDF3‐Mut) YTHDF3 constructs. l) Western blot demonstrating the expression of HELLS in HEK293T cells transfected with empty vector or wild‐type or mutant Flag‐tagged YTHDF3 plasmid. GAPDH was used as the negative control. The left panel presents a representative Western blot image. The right panel shows the quantification of the HELLS protein level. The average intensity of the NC group was set as 1.0. Each dot represents a single biological replicate. *p*‐Value was calculated with two‐tailed Mann–Whitney test. m) HEK293T cells were cotransfected with NC, YTHDF3‐WT, or YTHDF3‐Mut plasmids, and luciferase reporter plasmids carrying the HELLS 3’UTR, and luciferase activity was measured. *p‐*Value was calculated with two‐tailed Mann–Whitney test. TE, translational efficiency. FC, fold change. HOMER, Hypergeometric Optimization of Motif EnRichment. RIP, RNA immunoprecipitation. YTHDF3‐KD, YTHDF3 knockdown. NC, negative control. Ns, no significant difference. *p < 0.05, **p < 0.01, ***p < 0.001, ****p < 0.0001.

As we have mentioned above, m6A is critical to the downregulation of RNA TE in aged mouse oocytes; hence, we further analyzed the role of m6A in mediating RNA TE upon YTHDF3 depletion. Interestingly, we found that the TE of m6A‐enriched RNA was significantly decreased when YTHDF3 was knocked down (**Figure** **5**a,b; Figure S6a,b, Supporting Information). In addition, m6A‐enriched RNA occupied a larger proportion of YTHDF3‐KD‐affected high‐TE RNA (Figure S6c, Supporting Information). The amount of high‐TE RNA with m6A modification was drastically decreased when YTHDF3 was deficient in oocytes (Figure S6d, Supporting Information). Strikingly, RNA carrying papCPE or CPE showed strong repression in the TE of YTHDF3‐depleted oocytes (Figure S6e,f, Supporting Information). The repressive effects of papCPE or CPE were also significantly correlated with m6A modification (Figure S6g,h, Supporting Information). These results support the notion of m6A playing a vital role in modulating RNA TE upon YTHDF3 depletion.

It is well known that YTHDF3 facilitates RNA translation by binding m6A‐methylated transcripts.^[^ ^37^, ^65^ ^]^ We thus performed RNA immunoprecipitation sequencing (RIP‐seq) with an antibody specific to YTHDF3 in YTHDF3‐overexpressing HEK293T cells. There were 5758 overlapping potential candidate targets of YTHDF3 in three repeats (Figure 5c), most of which were enriched in 3’UTR sites (Figure S6i, Supporting Information). Functional enrichment analysis showed that distinct gene clusters, including “translation,” “regulation of RNA stability,” and “cell cycle,” were enriched in YTHDF3‐binding genes (Figure S6j, Supporting Information). Moreover, Hypergeometric Optimization of Motif EnRichment (HOMER) analysis identified an m6A consensus motif GGACU in the YTHDF3‐RIP peaks (Figure 5d). Next, we confirmed the role of YTHDF3 in regulating RNA TE by analyzing the YTHDF3‐RIP gene set. Interestingly, the TE of YTHDF3‐binding RNA was obviously decreased upon YTHDF3 depletion (Figure 5e; Figure S6k, Supporting Information). Next, we compared and identified overlap in m6A‐enriched genes from mouse (m6A‐Atlas) and human (RMBase) databases and the YTHDF3‐binding genes in HEK293T cells. This revealed that most of the genes bound by YTHDF3 were tagged with m6A, both in mice and humans (Figure S6l, Supporting Information). Of note, GSEA demonstrated that the RNA TE of m6A‐enriched and YTHDF3‐binding genes was much more downregulated when YTHDF3 was deficient (Figure 5f). These findings support the notion of YTHDF3 depletion predominantly downregulating TE in an m6A‐dependent manner, which may play a key role in translation inhibition of aging mouse oocytes.

### 2.7 HELLS is a Key M6A‐Modified YTHDF3‐Binding Target in Aged Mouse Oocytes

To identify the downstream m6A‐modified targets of YTHDF3 in aged mouse oocytes, we conducted integrative analysis by assessing the overlap in the differential TE genes, which were modified by m6A and bound by YTHDF3, of aged mouse oocytes and YTHDF3‐depleted oocytes (Figure 5g) and selected 449 candidate genes. Interestingly, the TE of 302 (67%) overlapping genes was decreased in both YTHDF3‐KD oocytes and aged mouse oocytes (Figure 5h). Next, to further narrow down the list, we analyzed the translatomics log2 fold change (FC) of these 302 genes in YTHDF3‐KD oocytes and aged mouse oocytes. The scatter plot showed the *Pearson* correlation coefficient in gene translational log_2_ FC between YTHDF3‐KD/control oocytes and aged/young oocytes was 0.2, with a *p*‐Value < 0.001, indicating slightly positively correlated (Figure S7a, Supporting Information). Notably, the translational levels of 36 genes were both downregulated (log2 FC<‐0.58) in YTHDF3‐KD oocytes and aged mouse oocytes (Figure 5i). Among the 36 identified genes (Table S2, Supporting Information), *Hells* has been suggested as an important chromatin remodeler that regulates meiotic kinetochore function and DNA double‐strand break repair, which plays critical roles in oocyte meiosis.^[^ ^66^, ^67^, ^68^ ^]^ Immunofluorescence confirmed that the expression of HELLS was decreased in YTHDF3‐KD mouse oocytes (Figure 5j). We found that the expression of HELLS in GV‐stage oocytes was distributed in both the cytoplasm and nucleus, and that YTHDF3 intervention knocked down the overall level of HELLS expression, including the expression in the nucleus (Figure 5j). In addition, to confirm the m6A modification of *Hells* in mouse oocytes, we performed methylated RNA immunoprecipitation quantitative polymerase chain reaction (MeRIP‐qPCR) with mouse oocytes (50 GV oocytes). The results showed that m6A was enriched at the 3’UTR of mouse *Hells* gene (Figure S7b, Supporting Information). MeRIP‐qPCR with HEK293T cells further confirmed that *HELLS* was modified by m6A in human cells as well (Figure S7c, Supporting Information). Additionally, *HELLS* was enriched in YTHDF3‐RIP peaks of HEK293T cells, the binding sites of which were predominantly distributed in the 3’UTR, coinciding with the distribution pattern of m6A along the length of mRNA (Figure S7d, Supporting Information). RIP‐qPCR in HEK293T cells verified that *HELLS* is a direct target of YTHDF3 (Figure S7e, Supporting Information).

To determine whether YTHDF3 regulates the translation of the *Hells* gene in an m6A‐dependent manner, we next generated a YTHDF3 mutant (YTHDF3‐Mut) by mutating the hydrophobic residues W438 and W492 to alanine (Figure 5k), which blocked the binding of YTHDF3 to the m6A site according to a previous study.^[^ ^69^ ^]^ The flag‐wild‐type YTHDF3 (YTHDF3‐WT) and YTHDF3‐Mut plasmids were overexpressed in HEK293T cells. Western blot analysis confirmed that YTHDF3 augmented the expression of the HELLS protein in an m6A‐dependent manner (Figure 5l), while the RNA level of *HELLS* remained unchanged (Figure S7f, Supporting Information), supporting the regulatory role of m6A‐dependent YTHDF3 in the RNA TE of *HELLS*. Next, we conducted a dual‐luciferase assay to analyze whether YTHDF3 regulated the translation of *HELLS* via m6A modification. Luciferase reporter genes with the *HELLS* 3’UTR were constructed and transfected into HEK293T cells. We found that wild‐type YTHDF3 overexpression largely enhanced the luciferase activity of the *HELLS* reporter, whereas mutated YTHDF3 could not increase the luciferase activity of the *HELLS* reporter (Figure 5m). These results suggest that YTHDF3 binds to the *HELLS* 3’UTR and promotes the translation of *HELLS* in an m6A‐dependent manner.

### 2.8 The Transcriptional and Translational Landscapes of Aged Human Oocytes

**Figure 6:**
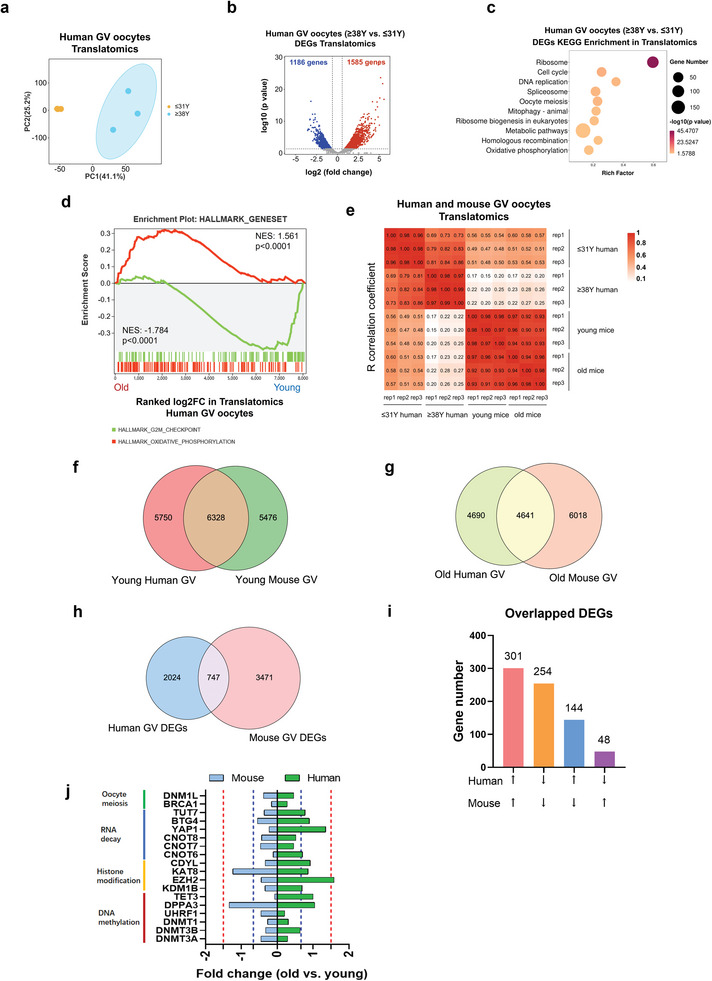
The transcriptional and translational landscapes of aged human oocytes. a) PCA plot of the translatomics of oocytes from young and aged human females. b) Volcano diagram showing DEGs detected by ultrasensitive translatomics. Red and blue dots denote up‐ and down‐regulated genes, respectively. *p* < 0.05, FC>1.5 or <0.67. c) Representative KEGG enrichment of DEGs identified by translatomics in aged human oocytes. d) Gene set enrichment analysis of the translatomics showing the translationally downregulated genes enriched in the hallmark of the G2/M checkpoint and translationally upregulated genes enriched in the hallmark of oxidative phosphorylation. e) Heatmap depicting the Pearson correlation coefficient of the translatome between each biological replicate from young and aged mice/humans. f) Venn plot of detected genes generated by single‐cell translatomics (TPM>1 for any biological replicate) in young human oocytes (≤31 years old) and in young mouse oocytes (≈4 weeks old). g) Venn plot of detected genes generated by single‐cell translatomics (TPM>1 for any biological replicate) in aged human oocytes (≥38 years old) and in aged mouse oocytes (>16 months old). h) Venn diagram showing the overlap of DEGs in aged mouse/human GV oocytes detected from the ultrasensitive translatomics (FC<0.67 or >1.5). i) The numbers of overlapped DEGs in aged human and aged mouse oocytes. j) The fold changes of specific genes in aged human and aged mouse oocytes. PCA, principal component analysis. FC, fold change. TPM, transcripts per million. DEGs, differentially expressed genes. KEGG, Kyoto Encyclopedia of Genes and Genomes.

As the reproductive aging of female humans occurs far earlier than that of mice, we next evaluated the transcriptional and translational profiling in human oocytes. The GV stage oocytes donated by young (≤31‐year‐old) and aged women (≥38‐year‐old) were conducted with single‐cell translatomics and single‐cell transcriptomics, respectively. Three biological replicates were generated, which showed high reproducibility (Figure S8a,b, Supporting Information). PCA analysis exhibited greater distinct clusters between young and aged oocytes in translatomics (**Figure** **6**a) than in transcriptomics (Figure S8c, Supporting Information). We found there were 1186 genes significantly downregulated and 1585 genes upregulated in translatomics (Figure 6b), while 224 genes were decreased and 189 genes increased in transcriptomics (Figure S8d, Supporting Information). Intriguingly, KEGG analysis demonstrated the differentially translated genes were enriched in “ribosome”, “oocyte meiosis”, and “homologous recombination” pathways (Figure 6c). Consistent with aged mouse oocytes, GSEA illustrated the upregulated genes of aged human oocytes in translatomics were enriched in oxidative phosphorylation, whereas the downregulated genes were correlated to G2M checkpoint (Figure 6d).

Mouse models are widely used to study the mechanisms of oocyte aging. However, there are huge differences between the onsets and the persistence of reproductive aging between the two species. Hence, to compare the conservations and the differences between human and mouse oocyte aging, integrative analyses were next conducted on translatomics of aged mouse and aged human oocytes. Intriguingly, we found a relatively higher correlation between young human oocytes and young mouse oocytes, whereas a very poor correlation between aged human oocytes and aged mouse oocytes (Figure 6e). There were 6328 (52.4% of young human GV translatome) and 4641 (49.7% of aged human GV translatome) genes commonly translated in young and aged GV oocytes of humans and mice, respectively. However, only 747 DEGs (26.9% of human DEGs) of young versus old were found between species (Figure 6f‐h). These results suggested less conservation in translational alterations than the translational expression between humans and mice during oocyte aging. However, interestingly, we found most of the overlapped DEGs exhibited the same expression trend in both species (Figure 6i). KEGG annotation indicated the overlapped upregulated genes were enriched in “ribosome”, “oxidative phosphorylation”, “splicesome”, and “ferroptosis”, while downregulated genes were correlated to “ubiquitin‐mediated proteolysis”, “cysteine and methionine metabolism”, and “cell cycle” pathways (Figure S8e,f, Supporting Information). Further, the expressions of specific genes (TPM>1) related to oocyte meiosis, RNA decay, histone modification, and DNA methylation were also evaluated (Figure 6j). Coincide with aged mouse oocytes, the translational levels of meiosis‐related genes *DNM1L* and *BRCA1*, and DNA methylation genes *UHRF1*, *DNMT1*, and *DNMT3A/B* were significantly decreased in aged human oocytes (Figure 6j). On the other hand, for RNA decay‐related genes, only the expressions of CNOT8 and CNOT7 were significantly reduced in human aging oocytes. Besides, different from aged mouse oocytes, there were no significant alterations of histone modification regulators in aged human oocytes (Figure 6j). These findings demonstrated the conservations and differences in oocyte aging between the two species, and addressed the role of RNA translational dysregulation in oocyte quality of aged humans.

### 2.9 RNA TE Dysregulation in Aged Human Oocytes is Irrelevant to M6A Modification

**Figure 7:**
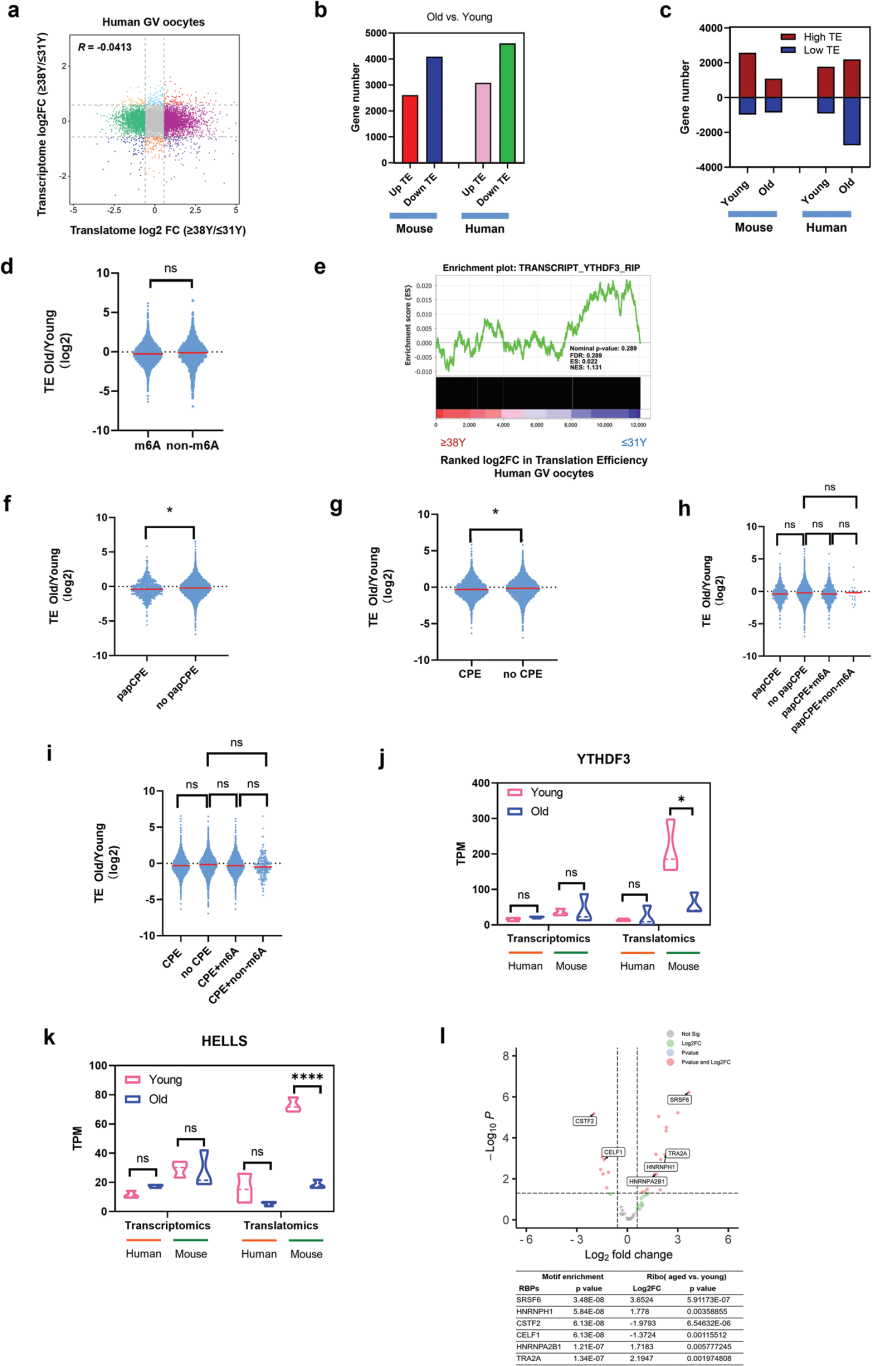
The changes of RNA translation efficiency in aged human oocytes. a) Scatter plot showing the changes in gene translation and transcription during oocyte aging. b) Bar plots showing the numbers of upregulated TE genes and downregulated TE genes in young and aged mouse/human GV oocytes, respectively. Upregulated, FC>1.5; downregulated, FC<0.67. c) Bar plots showing the numbers of high‐TE genes (TE>2) and low‐TE genes (TE<0.5) in young and aged mouse/human GV oocytes, respectively. d) Violin plots showing the TE changes in the genes of the group not enriched by m6A and of the m6A‐enriched group in aged human oocytes compared with young human oocytes. *p*‐Value was calculated with Student's t‐test for independent samples. e) Gene set enrichment analysis demonstrating the correlation of TE alterations and YTHDF3 target RNA in aged human oocytes. f) Violin plots showing the TE changes in genes of the papCPE‐containing group and in genes of the group not containing papCPE in aged human oocytes compared with young human oocytes. *p*‐Value was calculated with Student's t‐test for independent samples. g) Violin plots showing the TE changes in genes of the CPE‐containing group and in genes of the group not containing CPE in aged human oocytes compared with young human oocytes. *p*‐Value was calculated with Student's t‐test for independent samples. h) Violin plots showing the TE changes in four groups of genes in aged human oocytes compared with young human oocytes. *p*‐Values were calculated with one‐way ANOVA and Bonferroni post hoc test. i) Violin plots showing the TE changes in four groups of genes in aged human oocytes compared with young human oocytes. *p*‐Values were calculated with one‐way ANOVA and Bonferroni post hoc test. j) Transcriptional and translational expression levels of the *YTHDF3* in human/mouse GV oocytes. Data are shown as the mean ± SEMs. *p*‐Values were calculated with Student's t‐test for independent samples. k) Transcriptional and translational expression levels of the *HELLS* in human/mouse GV oocytes. Data are shown as the mean ± SEMs. p‐Values were calculated with Student's t‐test for independent samples. l) Representative RBPs enriched in 3’UTR of the genes in human oocytes that potentially regulated RNA TE. Data are shown as the means±SEMs. *p*‐Values were calculated with Student's t‐test for independent samples. FC, fold change. TE, translational efficiency. CPEs, cytoplasmic polyadenylation elements. papCPE, CPEs within 100 bp of PASs. RBPs, RNA binding proteins. Ns, no significant difference. *p < 0.05, **p < 0.01, ****p < 0.0001.

Similar to mouse oocytes, the RNA transcriptions and translations were nearly irrelevant in aged human oocytes, with a *Pearson* correlation coefficient of −0.0413 (**Figure** **7**a). Hence, to better understand the translation regulation, we next attempted to evaluate the TE alterations in aged human oocytes. Interestingly, similar to aged mouse oocytes, the TE of 2691 genes was increased and the TE of 4943 genes was decreased (Figure 7b). However, inconsistent with aged mouse oocytes, we noticed the downregulated genes in aged human oocytes were mainly low TE genes (Figure 7c). More strikingly, further analyses showed there was no significant difference in the TE between m6A‐ and non‐m6A‐enriched RNA (Figure 7d). Besides, YTHDF3‐targets were uncorrelated to the alterations of TE (Figure 7e). On the other hand, although RNA containing papCPE or CPE showed slight TE inhibition in aged human oocytes (Figure 7f,g). The RNA TE suppression with the presence of papCPE or CPE was hardly correlated to m6A modification (Figure 7h,i). Having demonstrated the vital role of YTHDF3 and HELLS in aged mouse oocytes, we found the translational levels of YTHDF3 and HELLS were relatively low in human oocytes. In addition, although we have observed significantly reduced translational levels of *Ythdf3* and *Hells* in aged mouse oocytes, there were no significant alterations in aged human oocytes (Figure 7j,k). These findings suggested a unique pattern of translational expression and regulation in aged human oocytes.

It has been illustrated that the translational expression in oocytes is mediated by translational regulatory elements bound by RNA‐binding proteins (RBPs) to the 3’UTR of the genes. Hence, we attempted to identify the potential RBPs that regulated RNA translation in aged human oocytes. A total of 109 RBPs were identified enriched in 3’UTR of the genes in human oocytes. Of these, 30 RBPs were differently expressed between aged and young oocytes (Table S3, Supporting Information). Therefore, we suggested that genes with altered translation efficiency in aged human oocytes may be related to the differently expressed RBPs, especially alternative splicing factors, such as SRSF6, HNRNPH1, CSTF2, and CELF1 (Figure 7l). However, the exact mechanisms of alternative splicing in regulating RNA translation in aged human oocytes still need further investigation.

## 3 Discussion

Considering the current social trend of delayed childbearing and the adverse fertility outcomes for aged women, there is a pressing need to elucidate the underlying mechanisms that reduce female oocyte quality and competence. As the cessation of gene transcription in fully grown GV oocytes occurs, the utilization of prestored proteins and the posttranscriptional modification/translation regulation of prestored RNA are essential events for oocyte meiotic maturation and OET. However, owing to the restrictions of cell number requirements in proteomics and ribosome profiling techniques, the protein expression and RNA translational landscapes in oocyte aging remain largely unknown. Here, we have taken advantage of the ultrasensitive translatomics/transcriptomics to document the translation patterns of mRNA in fully grown mouse/human oocytes during aging. The conservations and differences in gene expression between aged mouse and human oocytes were investigated. Additionally, we examined RNA degradation and epigenetic dynamics during oocyte aging. Our data shed light on the role of m6A‐mediated YTHDF3 in the translational regulation of aged mouse oocytes. Additionally, *Hells* was identified as a critical m6A‐dependent YTHDF3 target gene in regulating mouse oocyte quality during aging. However, the molecular basis underlying translational modulation in aged human oocytes needs further investigation.

In this study, we conducted ultrasensitive translatomics, transcriptomics, and single‐cell proteomics on aged and young mouse oocytes. Compared with single‐cell proteomics, which only detected ≈1200 proteins per single‐oocyte sample, ultrasensitive translatomics and transcriptomics provided a much higher coverage (nearly ninefold) of gene expression. Importantly, since gene transcription is silent in fully grown oocytes,^[^ ^25^ ^]^ the translatome is more accurate in reflecting changes in cellular events in oocytes. Consistent with reported gene expression patterns in oocytes, translatomics exhibited distinct PCA clusters of young and aged mouse oocytes and enabled the identification of DEGs that remained unchanged in transcriptomics.

It has been widely verified that aging oocytes are prone to meiotic chromosomal segregation errors, DNA damage and poor developmental potential.^[^ ^4^, ^52^, ^70^ ^]^ Our translatomics data in mouse and human oocytes enhance this understanding by showing that aberrant oocyte meiosis and homologous recombination are correlated with the dysregulation of translation. In particular, translational profiling revealed that mRNA coding for oxidative phosphorylation was translationally activated, and that mRNA required for the G2/M checkpoint of the cell cycle was mostly repressed either in aged mouse or in aged human oocytes. Noticeably, the translational expressions of oocyte‐meiosis‐related genes were globally reduced in aged mouse and human oocytes. On the other hand, our data also documented that the aged oocytes have less conservation between the two species, with only 26.9% of common DEGs. These data reminded us that the mouse is not a perfect model system to explore the mechanisms underlying oocyte aging in humans.

Previous studies have demonstrated the critical role of timely and efficient maternal RNA clearance in oocyte meiotic maturation, OET, and ZGA in the oocytes of both humans and mice.^[^ ^55^ ^]^ Meiotic maturation‐coupled mRNA degradation in oocytes is impaired with aging, causing negative biological consequences.^[^ ^6^ ^]^ Consistent with the findings of past studies, our study also illustrated that the translation of RNA‐decay regulators such as *Cnot6*, *Cnot6* *l*, and *Btg4* was repressed in aging mouse oocytes. However, human aging oocytes exhibit different translational patterns of RNA‐decay factors. For instance, although the translations of *CNOT7* and *CNOT8* were suppressed in human aging oocytes, the translation of *BTG4*, *YAP1*, and *CNOT6* remained constant. A rational explanation is the considerable individual differences among human oocytes, indicating the requirements of large‐scale experiments to further explore the characteristics of RNA degradome in human aging oocytes.

Epigenetic establishments are also crucial events during oogenesis, oocyte meiotic maturation, OET, and zygotic development.^[^ ^71^, ^72^ ^]^ Aberrant epigenetic dynamic modifications are associated with decreased female fertility in advanced maternal age.^[^ ^6^, ^14^, ^15^, ^16^, ^53^, ^73^ ^]^ Consistent with previous studies,^[^ ^17^, ^18^ ^]^ we reported the translational repression of DNA methylation factors, including *Dnmt1*, *Dnmt3a/b*, and *Uhrf1*, in aged mouse and human oocytes. Additionally, in aged mouse oocytes, the translation of factors associated with the establishment of H3K4me3, H3K27me3, H3K36me3, histone acetylation, and histone crotonylation was drastically decreased. However, these phenomena were variable in aged human oocytes. It is a technological challenge to investigate the epigenetic landscape in single oocytes, especially for scarce human oocytes. Hence, the translatomics method in this study is ideal for providing insight into the epigenetic dynamics of human and mouse oocytes during aging.

Previous studies have reported that aging is associated with ribosome pausing and a decreased translation rate, which is accompanied by aging‐related protein misfolding diseases.^[^ ^31^, ^32^ ^]^ However, the role of aberrant translational regulation in reproductive aging remains largely unknown. Of note, we discovered drastically high‐TE gene inhibition in aged mouse oocytes, which was associated with the cell cycle and chromosome organization. M6A is the most extensive RNA modification that regulates RNA translation in eukaryotes.^[^ ^37^ ^]^ Intriguingly, our analyses demonstrate that TE repression in aged mouse oocytes has a strong correlation with m6A modification. Notably, other researchers have documented that CPE or papCPE is essential to maintain translation repression.^[^ ^22^ ^]^ In this study, the integrative analyses strikingly showed that the repressive effects of papCPE were strongly related to m6A enrichment, which suggests that m6A may enhance the translationally repressive effects of papCPE, although deeper investigations and stronger evidence should be provided. These data reveal the crucial role of m6A in regulating TE repression in aged mouse oocytes. Shi et al.^[^ ^37^ ^]^ have addressed the functions of the m6A reader YTHDF3 in promoting translation in synergy with YTHDF1. Considering that the translational level of YTHDF3 is dramatically downregulated in aged mouse oocytes and that YTHDF3 depletion significantly reduced the in vitro PB1 emission rates of mouse oocytes, we suggest that YTHDF3 is pivotal in regulating translational repression in aged mouse oocytes. Translatomics demonstrated that the TE of genes, especially high‐TE genes, was drastically downregulated upon YTHDF3 depletion, which is similar to what occurs in aged mouse oocytes. Consistent with previous findings,^[^ ^69^ ^]^ our data revealed that YTHDF3 modulated the RNA TE in oocytes in an m6A‐dependent manner. Notably, a majority of m6A‐modified YTHDF3‐binding transcripts were compared and identified overlap between aged mouse oocytes and YTHDF3‐deficient oocytes, and the TE of most of these transcripts (67%) was drastically downregulated. Hence, our findings elucidate that m6A‐dependent YTHDF3 is a critical modulator of TE regulation in mouse oocyte aging.

Among the downstream genes of YTHDF3, we found that the translational levels and TE of the *Hells* gene in both aged mouse oocytes and YTHDF3‐KD oocytes were significantly downregulated. HELLS (encoded by *Hells*) protein is a member of the Snf2‐like chromatin remodeler family that is enriched at meiotic kinetochores.^[^ ^66^ ^]^ However, the role of HELLS in oocyte aging has not been reported. The crucial role of HELLS in maintaining centromere instability and abnormal chromosome segregation during oocyte meiosis has been indicated.^[^ ^66^ ^]^ In addition, HELLS facilitates homologous recombination and double‐strand break repair within heterochromatin.^[^ ^67^ ^]^ More importantly, HELLS is a crucial regulator of DNA methylation. In mouse embryonic stem cell and fibroblast models, HELLS facilitates de novo DNA methylation at repeat elements, transposons, and gene promoters by DNMT3A/3B.^[^ ^74^, ^75^ ^]^ Additionally, HELLS promotes DNA methylation by DNMT1 by enhancing UHRF1‐catalyzed histone H3 ubiquitination.^[^ ^76^ ^]^ As we have mentioned above, meiotic errors, DNA repair dysfunction, and reduced DNA methylation are crucial characteristics of aging oocytes. Hence, we suggest that *Hells*, one of the m6A‐containing YTHDF3 target genes, plays a key role in the quality/competence of aging oocytes by regulating chromosome segregation, DNA repair, and DNA methylation.

We have documented above that the translation of high‐TE genes was repressed in aged mouse oocytes, which was mediated by YTHDF3 in an m6A‐dependent manner. However, TE regulation in human oocytes is less conserved. Although the TE of genes was also downregulated in aged human oocytes, most of these downregulated genes were low‐TE genes. In addition, the TE of m6A‐enriched or YTHDF3‐target RNA in aged human oocytes remained constant, while the TE of pap‐CPE or CPE‐enriched RNA was slightly decreased. Besides, there were inapparent correlations between pap‐CPE or CPE and m6A modification. Although the role of YTHDF3 and HELLS was addressed in aged mouse oocytes, the expression of YTHDF3 or HELLS in aged human oocytes remained unchanged. These findings suggest a different TE regulation pattern in human oocytes. Functional studies in aging mouse models may not be sufficient and accurate for unveiling the gene expressions and molecular basis of human oocyte aging.

To better understand the disturbance of TE regulations in aged human oocytes, we identified 30 potential RBPs that regulated RNA translation in aged human oocytes. However, besides m6A, other translational regulators, including circle RNA, N4‐acetylcytidine, and alternative splicing complexes, may probably participate in oocyte development/aging.^[^ ^77^, ^78^, ^79^ ^]^ For instance, the functional annotations of DEGs in aged mouse or human oocytes have suggested the enrichment of RNA splicing pathways. In addition, we have observed the alteration of the alternative splicing complex EIF4A3.^[^ ^80^ ^]^ Hence, the mechanisms underlying RNA TE regulations in aging human oocytes need further investigation.

In summary, this study generated a single‐cell/ultrasensitive multi‐omics analysis in both aged and young mouse and human oocytes. We addressed the cross‐species conservations and differences between aged mouse and human oocytes in RNA translation. Additionally, we illustrated the role of dysregulated translation in mediating aberrant epigenetic establishment and abnormal maternal RNA clearance during oocyte aging. Aged mouse oocytes exhibited a globally downregulated TE, especially the high‐TE genes, which was related to decreased oocyte quality. Using a YTHDF3‐depleted oocyte model, we demonstrated the role of m6A‐mediated YTHDF3 in modulating RNA TE of aged mouse oocytes and identified a critical m6A‐dependent YTHDF3‐target gene, *Hells*. Many downregulated genes in aged humans have low‐TE, and m6A has little effect on TE regulation. Understanding the details of translational regulation in aged human oocytes via future investigations will be of great interest. This study provides novel insights into the molecular mechanisms underlying oocyte aging and suggests a new potential interventional strategy for improving aging oocyte quality.

## 4 Experimental Section

### Mice

Female 3‐week‐old and 8‐month‐old female KM mice were purchased from Vital River Laboratory Animal Technology Co., Ltd (Beijing, China). The 8‐month‐old female mice were raised in the specific pathogen‐free laboratory for more than 8 months. Before the experiment, the 3‐week‐old mice were acclimated to the laboratory environment for 1 week. The age of old and young mice used in this study was approximately 16 months old and 4 weeks old, respectively. The laboratory environment was maintained with a 12 h:12 h light/dark cycle, and the temperature (22–24 °C) and humidity (50–60%) were under control. The procedures of oocyte collecting were approved by the Animal Care and Use Committee of the Sixth Affiliated Hospital, Sun Yat‐sen University (Guangzhou, China), with the ethical approval numbers IACUC‐2021101102 and IACUC‐2020053102.

### Mouse GV Oocytes Collection

Female KM mice were intraperitoneally injected with 10U of pregnant mare serum gonadotropin (Ningbo Second Hormone Factory, Hangzhou, China). Forty‐six to 48 h later, ovaries were isolated and maintained in M2 medium (Sigma‐Aldrich, M7167). Cumulus‐oocyte complexes (COCs) were released from antral follicles by puncturing with a sterile needle. Cumulus cells were removed with hyaluronidase (Sigma‐Aldrich, 37326‐33‐3). Denuded GV oocytes were collected for further experiments.

### Human GV Oocytes Collection

Female patients, who were diagnosed with male factor infertility or tubal infertility, underwent intracytoplasmic sperm injection (ICSI) treatment from September 2021 to March 2022 were enrolled. They were conducted ovarian hyperstimulation with a short protocol of GnRH agonist. The follicles' size was evaluated via transvaginal ultrasonography. Triggered with 250 µg of HCG (Ovidrel, WD22‐354) when at least two leading follicles reached a diameter of 18 mm. COCs were collected with an oocyte retrieval needle under the guidance of transvaginal ultrasonography. Cumulus cells were denuded with hyaluronidase and repeated pipetting (Sigma‐Aldrich, 37326‐33‐3). The maturity of oocytes was accessed under a microscope. The MII oocytes were used for ICSI therapy. And the GV oocytes were washed with PBS 3 times, collected in 10 µL sample buffer (Vazyme, N712) and stored in liquid nitrogen for further single‐cell transcriptomes and translatomes sequencing (T&T‐seq). There were four GV oocytes collected form the young and the aged group, respectively, which were conducted T&T‐seq. The collection of GV oocytes was approved by the donors with the informed consents. All the procedures involving human oocyte collection were authorized by the Ethics Committee of The Sixth Affiliated Hospital of Sun Yat‐sen University, with a certificate number of 2021ZSLYEC‐512.

### In Vitro Maturation (IVM)

GV‐stage COCs were incubated in the TCM‐199 medium (Gibco; 31 100 035), supplemented with 10% fetal bovine serum (FBS) and 0.2 mm sodium pyruvate, which were cultured in an incubator with 5% CO_2_/95% air at 37 °C for 14 h. The cumulus cells were removed, and the denuded oocytes were observed under a microscope to record their maturity. The oocyte in vitro maturation rate was evaluated based on the first polar body (PB1 emission rate).

### Immunofluorescence Staining

Denuded oocytes were fixed in 4% paraformaldehyde (Servicebio; G1101) at room temperature for 30 min. The fixing solution was removed and incubated the oocytes in PBS with 0.2% Triton X‐100 at room temperature for 30 min to permeabilize. The oocytes were washed with 3% BSA in PBS three times and then incubated them with 3% BSA at room temperature for 1 h. The oocytes were incubated with primary antibody (diluted with 3% BSA, 1:200) at 4 °C, and placed them in a wet box overnight. The oocytes were washed with 0.3% BSA three times, and incubated in a secondary antibody (diluted with 3% BSA, 1:500) at room temperature for 1 h, which was then placed in a wet box out of light. For DAPI counterstain in the nucleus, the oocytes were washed with 0.3% BSA three times and then incubated with DAPI solution at room temperature for 15 min, out of light. The oocytes were washed with 0.3% BSA three times, and placed them in 20–30 µL 0.3% BSA at room temperature, out of light. The oocytes were observed and pictures were captured under an inverted confocal microscope. Antibodies used in this study are listed as follows: YTHDF3 (Novus Biologicals, 94636), HELLS (Proteintech, 11955‐1‐AP),

### Electroporation

Denuded mouse GV oocytes were collected as described above. Incubated the denuded GV oocytes in the Tyrode's solution (Leagene, CZ0060) for 10 s to weaken the zona pellucida. The oocytes were washed in Opti‐MEM medium (Gibco, 31985‐062) three times. All antibodies were concentrated using Amicon Ultra‐0.5 100 KDa centrifugal filter devices (Millipore) to remove traces of azide and replace the buffer with PBS. For the concentration of the antibody, different concentrations for each antibody were tested, and the results showed that a concentration of 10–200 ng µL^−1^ would be effective. The antibody was diluted in OPTI‐MEM and mixed it with 200 ng µL^−1^ trim21 mRNA. The oocytes with 5 µL diluted antibody were transferred to the flat electrode chamber of the Pulse Generator CUY21EDIT II (BEX Co., Ltd., Tokyo, Japan). Electroporation was executed with the parameters of 30 volts in amplitude, 1 ms pulse width, and 4 pulses at intervals of 50 ms. The oocytes were washed with Opti‐MEM medium three times, and incubated in Opti‐MEM medium at 37 °C with 5% CO_2_/95% air for 30 min. Further, the oocytes were transferred into 3‐isobutyl‐1‐methyl‐xanthine (IBMX, MCE, HY‐12318)‐containing IVM medium (50 µm IBMX) at 37 °C with 5% CO_2_/95% air for 6 h. The GV oocytes were then washed with IBMX‐free IVM medium three times. The GV oocytes were collected for IVM or further experiments. The antibodies used in electroporation were as follows: YTHDF3 (Abcam, ab220161), HELLS (Proteintech, 11955‐1‐AP), WTAP(Proteintech, 10200‐1‐AP), YTHDC1(Novus biologicals, NBP1‐81353), YTHDF1(Proteintech, 17479‐1‐AP), YTHDF2(Novus biologicals, NBP2‐58340), ALKBH5(Proteintech, 16837‐1‐AP).

### Ultrasensitive Transcriptomics and Translatomics Sequencing (T&T‐Seq)

The procedures of ultrasensitive T&T‐seq were conducted as a previous study described. For mouse oocytes, each sample had four oocytes. Oocytes were lysed in 10 µL sample buffer consisting of 9 µL lysis buffer and 1 µL RNase inhibitor (Vazyme, n712) on ice for 10 min. The lysates were mixed and divided into two parts, of which 1.5 µL lysates were used for transcriptome and 8.5 µL for translatome. For translatome, the RiboLace beads (Immagina, RL001) were functionalized according to the manufacturer's instructions. And then, the functional RiboLace beads were divided into 10 µL each sample with a 1.5 mL EP tube. The solution of functional RiboLace beads was removed. The functional RiboLace beads were resuspended with 8 µL lysates, 1 µL RNase inhibitor, and 8 µL binding buffer (RNase free water supplemented with 100 µg µL^−1^ cycloheximide, 1 mm DTT, 5 mm MgCl_2_, 150 mm NaCl, and 20 mm Tris‐Hcl pH 8.0), and incubated on a rotator at 3 rpm at 4 °C for 1 h. The solution was discarded and the beads were washed with W‐buffer (Immagina, RL001) on a magnet twice. The beads were resuspended with 12 µL RLT (Qiagen,74 004) supplemented with 10% of beta‐mercaptoethanol and 1% of glycoblue. Incubated at room temperature for 5 min, collected the solution to a new PCR tube, and discarded the beads. Fifteen microliters 2 m LiCl and 54 µL VAHTS RNA Clean Beads (Vazyme, N412) were added to the solution described above. The ribosome binding full‐length RNA was isolated and purified according to the manufacturer's instructions. Both total RNA (the 2 µL lysates described above) and ribosome binding full‐length RNA were reverse transcribed to cDNA and amplified for 18 PCR cycles, according to the protocol of Single Cell Full Length mRNA‐Amplification Kit (Vazyme, n712). VAHTS DNA Clean Beads (Vazyme, N411) were used to purify the cDNA amplification products. Further, the concentration of cDNA was assessed by Qubit (Invitrogen, USA), and the peak was detected by Bioanalyzer 2100 (Agilent, CA, USA), which was ≈2000 bp. The indexed libraries were constructed using TruePrep DNA Library Prep Kit V2 for Illumina (Vazyme, TD502). VAHTS DNA Clean Beads (Vazyme, N411) were used to fractionate and purify the amplified product to 250–450 bp. The quantities of libraries were identified by Qubit (Invitrogen, USA) and Bioanalyzer 2100 (Agilent, CA, USA). Pair‐end sequencing was conducted with Illumina Novaseq 6000 platform (Azenta Life Sciences, Hangzhou, China), with the sequencing mode of PE150.

### Single‐Cell Proteomics

Protein Extraction: Samples were sonicated three times on ice by a high‐intensity sonicator (Scientz) in a lysis buffer containing 8 m urea and 1% protease inhibitor. The remaining debris was removed by centrifugation at 12 000 × *g* for 10 min at 4 °C. Finally, the supernatant was collected for the determination of protein concentration by using the BCA kit according to the manufacturer's instructions.

Trypsin Digestion: The protein was digested as follows: 1) Reduction: 5 mm dithiothreitol for 30 min at 56 °C; 2) Alkylation with 11 mm iodoacetamide for 15 min at room temperature in the dark; 3) Use Dilute protein samples to a urea concentration of less than 2 m with 100 mm TEAB; 4) Add trypsin at a mass ratio of 1:50 to digest overnight; 5) Use 1:100 trypsin to digest again for 4 h; 6) Use C18 SPE column to purify peptides. Finally, iodoacetamide was added to make the final concentration of the mixed samples 11 mm and incubated at room temperature for 15 min in the dark.

LC‐MS Analysis: The peptides were dissolved in phase A of the liquid chromatography mobile phase (containing 0.1% formic acid and 2% acetonitrile) and separated using an EASY‐nLC 1000 ultra‐high performance liquid phase system. Mobile phase B was a solution containing 0.1% formic acid and 100% acetonitrile. Liquid gradient settings: 0–4 min, 7–16%B; 4–12 min, 16–26%B; 12–15 min, 26–40%B; 15–17 min, 40–80%B; 17–20 min, 80%B; 20–23 min, 80–30%B; 23–26 min, 30%B; 26–28 min, 30–80%B; 28–30 min, 80%B, the flow rate was maintained at 100 nL min^−1^. The peptides were separated by an ultra‐high performance liquid phase system and injected into the Capillary ion source for ionization at 1.75 kV. The product was then analyzed using timsTOF Pro mass spectrometry. The peptide precursor ions and their secondary fragments were detected and analyzed using high‐resolution TOF, and the scanning range of the secondary mass spectrometer was set to 100–1700. Parallel accumulation serial fragmentation (PASEF) mode was used for data acquisition. After a first‐order mass spectrometer was acquired, three times of PASEF modes were used to collect the second‐order spectrum with the charge number of the precursor ion in the range of 0–5, and the dynamic exclusion time of the tandem mass spectrometer scan was set to 30 s to avoid repeated scanning of the precursor ion.

Database Search: MS/MS data were processed using the MaxQuant search engine (v.1.6.15.0). Tandem mass spectrometry was searched based on the human SwissProt database (20 422 entries). In the first search, the mass tolerance of the precursor ions was set to 20 ppm, followed by the main search with a mass tolerance of 5 ppm and a fragment ion of 0.02 Da. The fixed modification was set to the carbamoylmethyl group on Cys. Variable modifications were set to acetylation at the N‐terminus of the protein and oxidation on Met. FDR adjusted to < 1%.

Enrichment analysis: Differentially expressed proteins were enriched based on GO and KEGG database using a two‐tailed *Fisher's* exact test for all identified proteins, with a corrected *p*‐Value < 0.05.

### RIP Sequencing and qPCR

The human embryonic kidney HEK293T cells (FuHeng Biology, FH0244) were cultured with high‐glucose DMEM medium (Gibco, C11960500BT) containing 1% penicillin‐streptomycin and 10% FBS at 37 °C with 5% CO_2_/95% air. The YTHDF3‐overexpressing plasmids (GeneCopeia, EX‐H0962‐M12) were transfected into HEK293T cells following the manufacturer's instructions of Lipofectamine 3000 Transfection Reagent (Thermo Fisher Scientific, L3000001). The cells were washed with cold PBS for one time. One milliliter purification buffer, 5 µL NP40, and 1 µL protease inhibitor (APExBIO, K1007) were added to resuspend the cell sediment and lysed on ice for 5 min. Centrifuged at 2000 rpm at 4 °C for 10 min. One milliliter lysate was collected to a new 1.5 mL EP tube, which could be stored at −80 °C.

For the input group, the RNA of 100 µL cell lysates was purified following the protocol of the RNeasy MinElute Cleanup Kit (Qiagen, 74 204) and the RNA was eluted by 20 µL RNase free water.

For the RIP groups, the A/G beads (MCE, HY‐K0202) were functionalized with a purification buffer. The functional A/G beads were incubated with YTHDF3 antibody (Abcam, ab220161) or IgG antibody (FineTest, FNSA‐0106) at a rotator at room temperature for 2 h. The beads were washed with purification buffer twice and the solution was discarded. Four hundred fifty microliters of HEK293T cells lysates, which were described above, 1 µL RNase inhibitor (APExBIO, K1046), and 0.25 m EDTA were added to the YTHDF3 conjugated and IgG conjugated A/G beads, respectively. Incubated at a rotator at 4 °C for 4 h. The supernatant was removed and washed with purification buffer three times. The beads were resuspended with 117 µL lysis buffer, 18 µl 10 mg mL^−1^ proteinase K, and 15 µL 10% SDS, and incubated for 30 min at 55 °C in a shaker at 200–300 rpm. The supernatant was transferred to a new 1.5 mL EP tube. The beads were washed with 250 µL purification buffer and the solution was collected to the 1.5 mL EP tube described above. Four hundred microliters of phenol chloroform isoamyl alcohol was added and vortexed for 15 s. Centrifuged for 10 min at room temperature at 14 000 rpm and transferred 300 µL aqueous phase to a new 1.5 mL EP tube. Thirty microliters NaAc and 825 µL ethanol were added and stored at −80 °C for 3 h. Centrifuged for 30 min at 4 °C at 14 000 rpm, removed the supernatant gently. The sediments were washed with 1 mL 80% ethanol, centrifuged for 15 min at 4 °C at 14 000 rpm, and removed all the solution gently. The sediments were uncovered and dried for 5 min. The RNA was eluted with 20 µL RNase‐free water.

### qPCR

For qPCR, the purified RNA was synthesized as cDNA via reversed transcription according to the guidance of HiScript III RT SuperMix for qPCR (Vazyme, R323‐01). 2× RealStar Green Power Mixture (Genstar, A311‐101) and Roche LightCycler 480 II (Roche Diagnostics, Germany) were used to carry out qPCR.

### MeRIP

MeRIP‐seq was performed according to a previously reported protocol.^[^ ^81^ ^]^ Briefly, after total RNA extraction using Qiagen RNAeasy micro kit, the product was fragmented and purified. A portion of the original fragmented mRNA was kept as input. Subsequently, a portion of the fragmented mRNA was bound to protein A/G magnetic beads in a purification buffer containing anti‐m 6 A antibody (Abcam, GR3285732) and incubated at 4 °C for 4 h. Proteinase K buffer (APEXBIO, K1037) was used to digest antibodies to purify methylated RNA. The purified RNA was synthesized as cDNA and detected by qPCR.

For ultrasensitive MeRIP in oocytes, the MeRIP procedure was optimized as follows. Protein A coupled with Halotag was purified using Halotag beads (Progema, G7281). 2.5 µg of anti‐m6A antibody was incubated with 50 µL of protein A‐Halotag beads in the purification buffer with rotation for 2 h at room temperature. After incubation, the beads were washed with a purification buffer. Oocytes (50) were lysed in 25 µL purification buffer with 0.5% NP40 and 0.3% RNase inhibitor on ice for 20 min. The lysates were mixed and divided into two parts, of which 1.5 µL lysates were used for input and 23.5 µL for MeRIP. For MeRIP, the purified protein A‐m6A antibody compounds were incubated with 23.5 µL lysates at 1400 rpm for 2 h at room temperature. And then, the beads were washed with a purification buffer, and the solution of Halotag beads was removed. Resuspended the beads with 12 µL RLT (Qiagen,74 004) supplemented with 10% of beta‐mercaptoethanol and 1% of glycoblue. Incubated at room temperature for 5 min, collected the solution to a new PCR tube, and discarded the beads. Added 15 µL 2 m LiCl and 54 µL VAHTS RNA Clean Beads (Vazyme, N412) to the solution described above. Input RNA and MeRIP RNA were reverse transcribed to cDNA and detected by qPCR.

### Dual‐Luciferase Reporter Assay

Dual luciferase reporters were performed according to a previously reported protocol.^[^ ^82^ ^]^ Briefly, a YTHDF3 mutant plasmid, including amino acid 438 and amino acid 492 mutation sites, was constructed based on the EX‐H0962‐M12 plasmid. According to the sequence downloaded from the NCBI website, the 3′‐UTR of human HELLS was cloned into the rear end of the mutated YTHDF3 plasmid. Renilla/firefly luciferase activity values were measured using the Dual‐Luciferase Reporter Gene Assay Kit according to the manufacturer's instructions and then normalized to calculate relative luciferase activity.

### Western Blot

For the western blot, after lysing the samples in sodium dodecyl sulfate (SDS) sample buffer for 5 min, they were separated using SDS‐PAGE gels. After blocking the samples on polyvinylidene fluoride membranes with 5% low‐fat milk for 1 h, the samples were incubated with the antibody for Flag, HELLS, and GAPHD overnight at 4 °C. Add 1:5000 anti‐goat rabbit‐radish peroxidase‐conjugated secondary antibody to the product thoroughly washed with PBST and incubated for 1 h at room temperature. Detected Flag, HELLS, and GAPHD (normalized internal controls) were quantified using ImageJ software after processing with a chemiluminescence system.

### M6A and CPE /papCPE Annotation

The common m6A data of mice and humans were derived from the m6A‐Atlas database.^[^ ^83^ ^]^ CPEs and papCPEs data in 3′UTRs were derived from the CPE database^[^ ^84^ ^]^ (https://genome.crg.es/CPE/).

## Conflict of Interest

The authors declare no conflict of interest.

## Author Contributions

J.H. and P.C. contributed equally to this work as first authors. C.Z. designed the project and directed the research. J.H. carried out overall experiments and analyzed the data. J.H., P.C., L.J., T.L., X.Y., and W.H. collected the human oocytes and analyzed the bioinformatics data. Q.L. performed animal experiments and immunofluorescence assay. Y.Z. conducted immunofluorescence assay and electroporation. J.L. and T.W. carried out cellular experiments. W.H. and K.K. provided technical support for ultrasensitive transcriptional and translational dual‐omics. H.Z. and X.L. supervised the project. X.L. provided financial support. J.H. and P.C. wrote the original draft. C.Z., K.K., X.L., and H.Z. reviewed the writing. J.H. and C.Z. revised the paper. All authors read and approved the final manuscript.

## Supporting information

Supporting Information

## Data Availability

The raw sequence data reported in this paper have been deposited in the Genome Sequence Archive (Genomics, Proteomics & Bioinformatics 2021) in the National Genomics Data Center (Nucleic Acids Res 2022), China National Center for Bioinformation / Beijing Institute of Genomics, Chinese Academy of Sciences (GSA: CRA008819; GSA‐Human: HRA003403, HRA004706) that are publicly accessible at https://ngdc.cncb.ac.cn/gsa and https://ngdc.cncb.ac.cn/gsa‐human.
