# Supplementary material for: Multi‐Omics Analysis Reveals Translational Landscapes and Regulations in Mouse and Human Oocyte Aging
Source: Adv Sci (Weinh). 2023 Jul 3;10(26):2301538. doi: 10.1002/advs.202301538 (PMC10502832; doi:10.1002/advs.202301538)
Supplement: Supplementary file 1 — Supporting Information [file ADVS-10-2301538-s001.pdf]

ADVANCED SCIENCE
Open Access

Supporting Information

for *Adv. Sci.*, DOI 10.1002/advs.202301538

Multi-Omics Analysis Reveals Translational Landscapes and Regulations in Mouse and Human Oocyte Aging

Jiana Huang, Peigen Chen, Lei Jia, Tingting Li, Xing Yang, Qiqi Liang, Yanyan Zeng, Jiawen Liu, Taibao Wu, Wenqi Hu, Kehkooi Kee, Haitao Zeng*, Xiaoyan Liang* and Chuanchuan Zhou**

Supporting Information

Multi-Omics Analysis Reveals Translational Landscapes and Regulations in Mouse and Human Oocyte Aging

Jiana Huang#, Peigen Chen#, Lei Jia, Tingting Li, Xing Yang, Qiqi Liang, Yanyan Zeng, Jiawen Liu, Taibao Wu, Wenqi Hu, Kehkooi Kee, Haitao Zeng*, Xiaoyan Liang*, Chuanchuan Zhou**

This PDF file includes:

Figure. S1 to S8

Table. S1 to S3

Supplementary Figures:

Figure S1

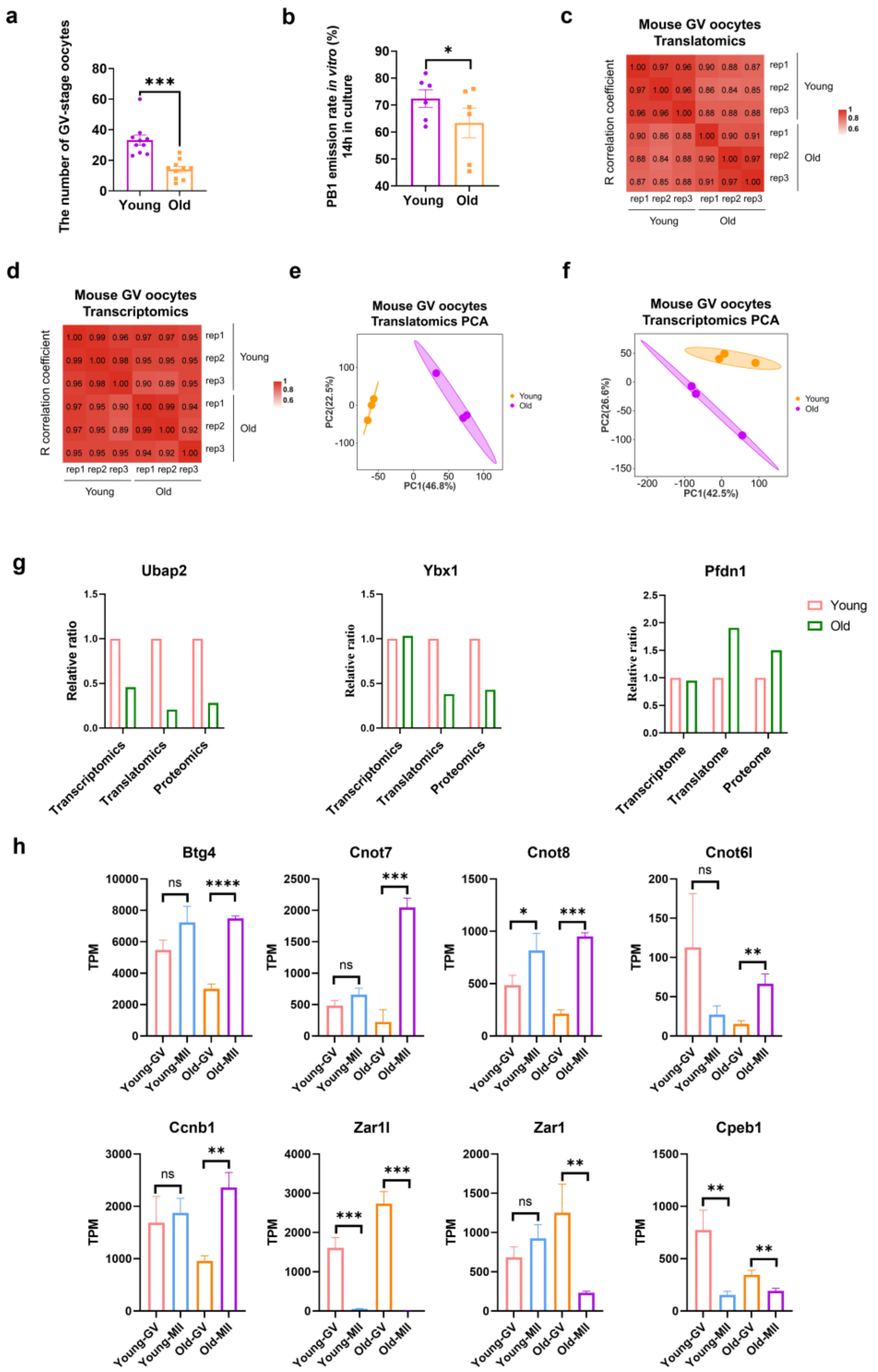

Figure S1. Multi-omics analyses of aged and young mouse oocytes. a) The number of germinal vesicle (GV)-stage oocytes retrieved from young and aged mice. Each dot represents a single biological replicate. p-Values were calculated with Student's t-test for paired samples. b) The in vitro PB1 emission rates of oocytes from young and aged

mice. Each dot represents a single biological replicate. p-Values were calculated with Student's t-test for paired samples. c) Heatmap depicting the *Pearson* correlation coefficient of the translatome between each biological replicate from young and aged mice. d) Heatmap depicting the *Pearson* correlation coefficient of the transcriptome between each biological replicate from young and aged mice. e) PCA plot of the translatomics of young and aged mouse GV oocytes. f) PCA plot of the transcriptomics of young and aged mouse oocytes. g) The transcriptional, translational, and protein levels of representative genes in young and aged mouse GV oocytes. h) The translational levels of representative genes in young and aged mouse GV and MII oocytes. PB1, polar body-1. PCA, principal component analysis. Ns, no significant difference. *P<0.05, **P<0.01, *** P<0.001, **** P<0.0001.

Figure S2

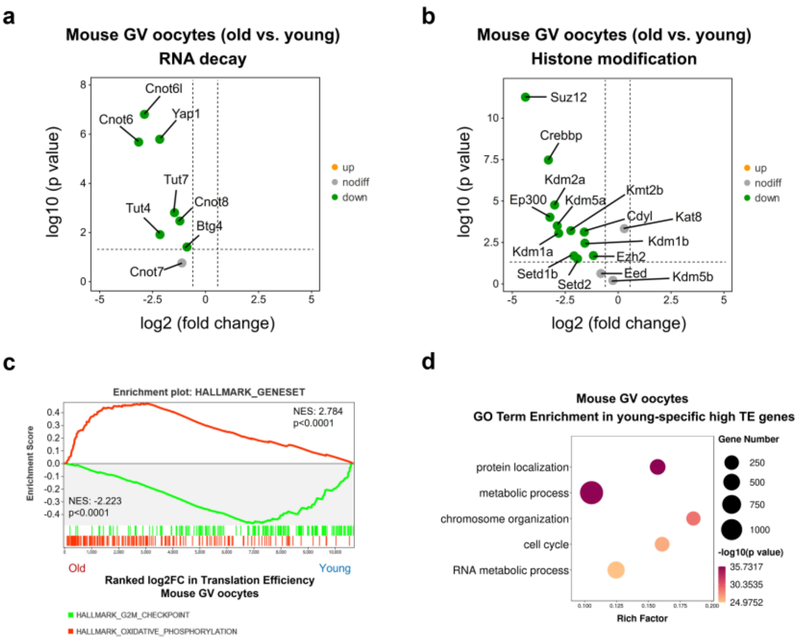

Figure S2. Translational efficiency of aged and young mouse oocytes. a) Volcano plot showing the changes in the RNA decay-related genes identified by translatomics. b) Volcano plot showing the changes in the histone modification regulatory genes identified by the translatomics. c) Gene set enrichment analysis of TE showing the downregulated genes enriched in the hallmark of the G2/M checkpoint and the upregulated genes enriched in the hallmark of oxidative phosphorylation. d) Gene ontology term enrichment of the young-specific high-TE genes of aged mouse oocytes. TE, translational efficiency.

Figure S3

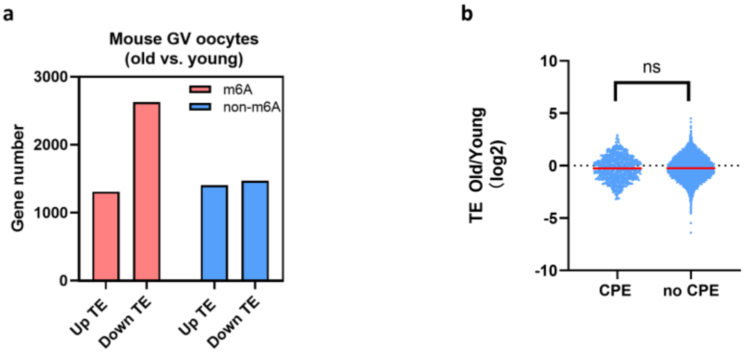

Figure S3. Correlations of translational efficiency changes and m6A modification/CPE existence in aged mouse oocytes. a) Bar plots showing the numbers of up- and down-regulated TE genes between aged and young mouse oocytes. Pink denotes the m6A-enriched genes. Blue denotes genes not containing m6A. b) Violin plots showing the changes in CPE-containing and non-CPE-containing genes in aged mouse oocytes. CPE, cytoplasmic polyadenylation element. TE, translational efficiency. *p*-Value was calculated with Student's t-test for independent samples. Ns, no significant difference.

Figure S4

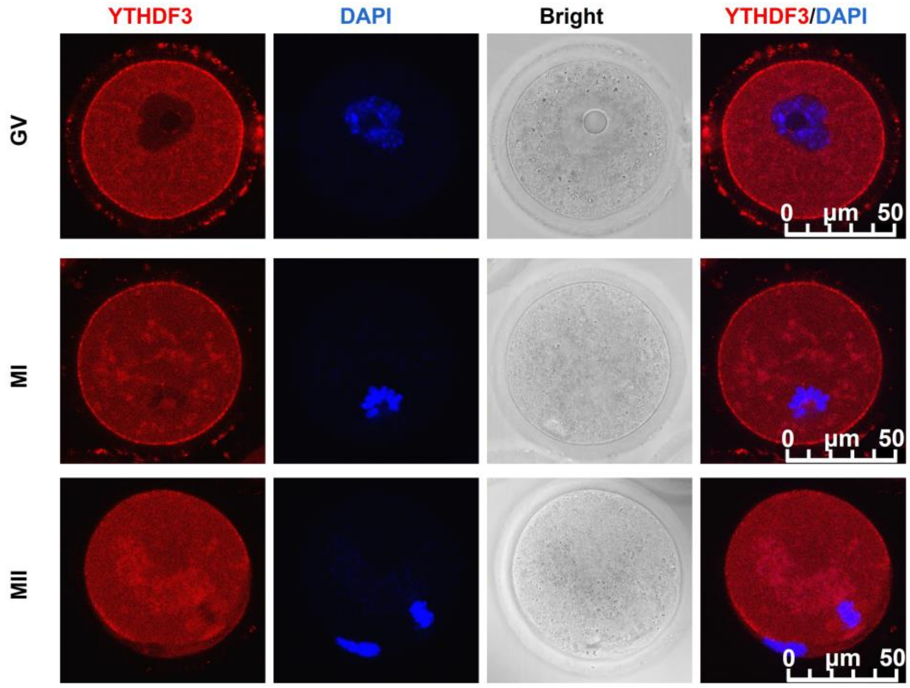

Figure S4. Immunofluorescence verifying the expression of YTHDF3 in young mouse

oocytes during in vitro maturation. Scale bar, 50 µm.

Figure S5

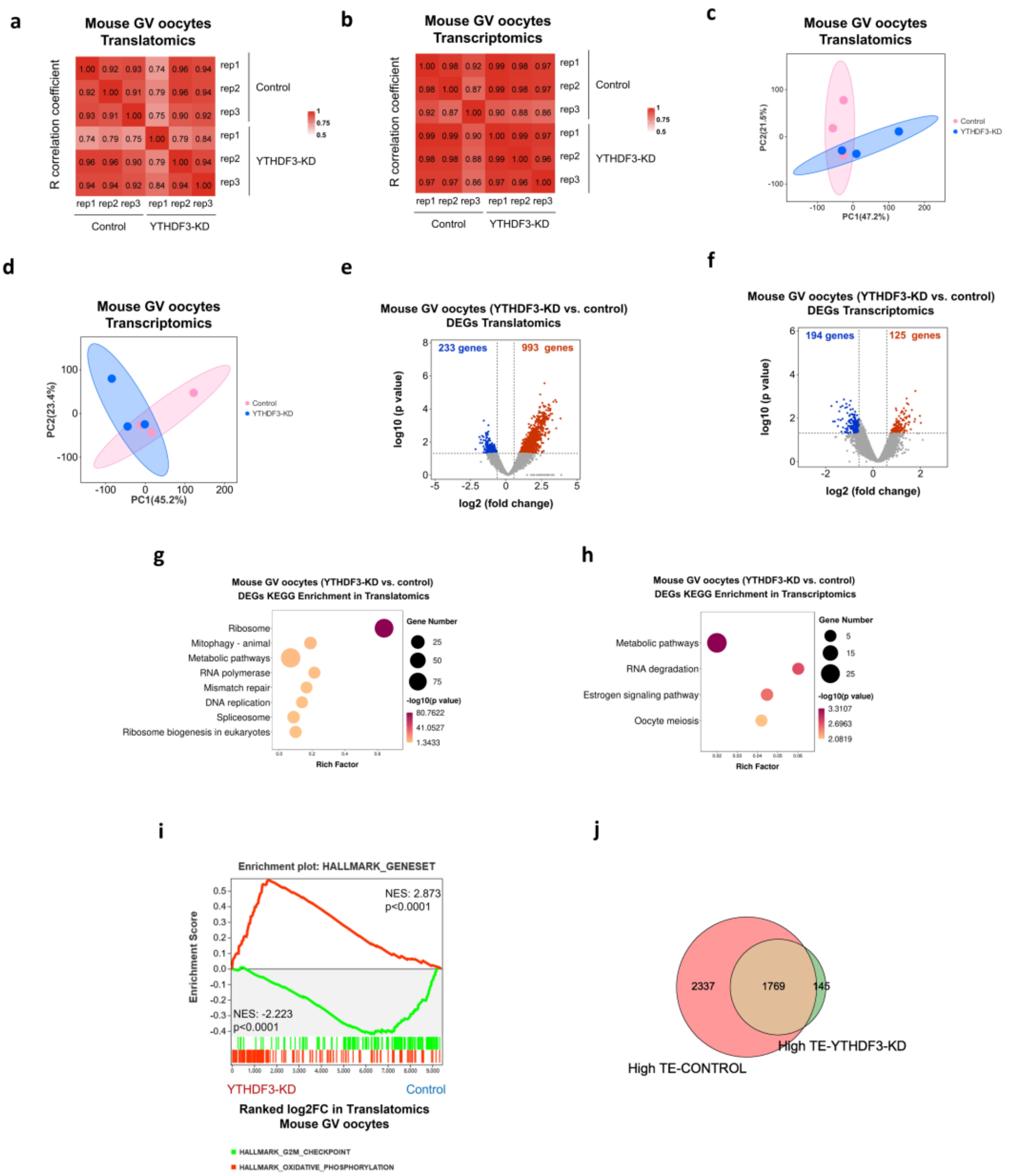

Figure S5. The translational and transcriptional landscapes of oocytes upon YTHDF3 depletion. a) Heatmap depicting the *Pearson* correlation coefficient of the translatomics between each biological replicate from the control group oocytes and the YTHDF3-KD group oocytes. b) Heatmap depicting the *Pearson* correlation coefficient of the transcriptome between each biological replicate from the control group oocytes and the YTHDF3-KD group oocytes. c) PCA plot of the translatomics in oocytes from the control group and the YTHDF3-KD group. d) PCA plot of the transcriptomics in oocytes from the control group and the YTHDF3-KD group. e,f) Volcano diagram showing DEGs detected by the single-cell translatomics (e) and transcriptomics (f). Red

and blue dots denote up- and down-regulated genes, respectively. $P<0.05$, FC>1.5 or <0.67. g,h) KEGG analysis of the DEGs detected by translatomics (g) and transcriptomics (h) analyses. i) Gene set enrichment analysis of the translatomics showing the translationally downregulated genes enriched in hallmarks of the G2/M checkpoint and translationally upregulated genes enriched in hallmarks of oxidative phosphorylation. j) Venn diagram showing the overlap of high-TE genes identified from the control group oocytes and the YTHDF3-KD group oocytes. YTHDF3-KD, YTHDF3 knockdown. PCA, principal component analysis. DEGs, differentially expressed genes. FC, fold change. KEGG, Representative Kyoto Encyclopedia of Genes and Genomes. High TE, translation efficiency > 2.

Figure S6

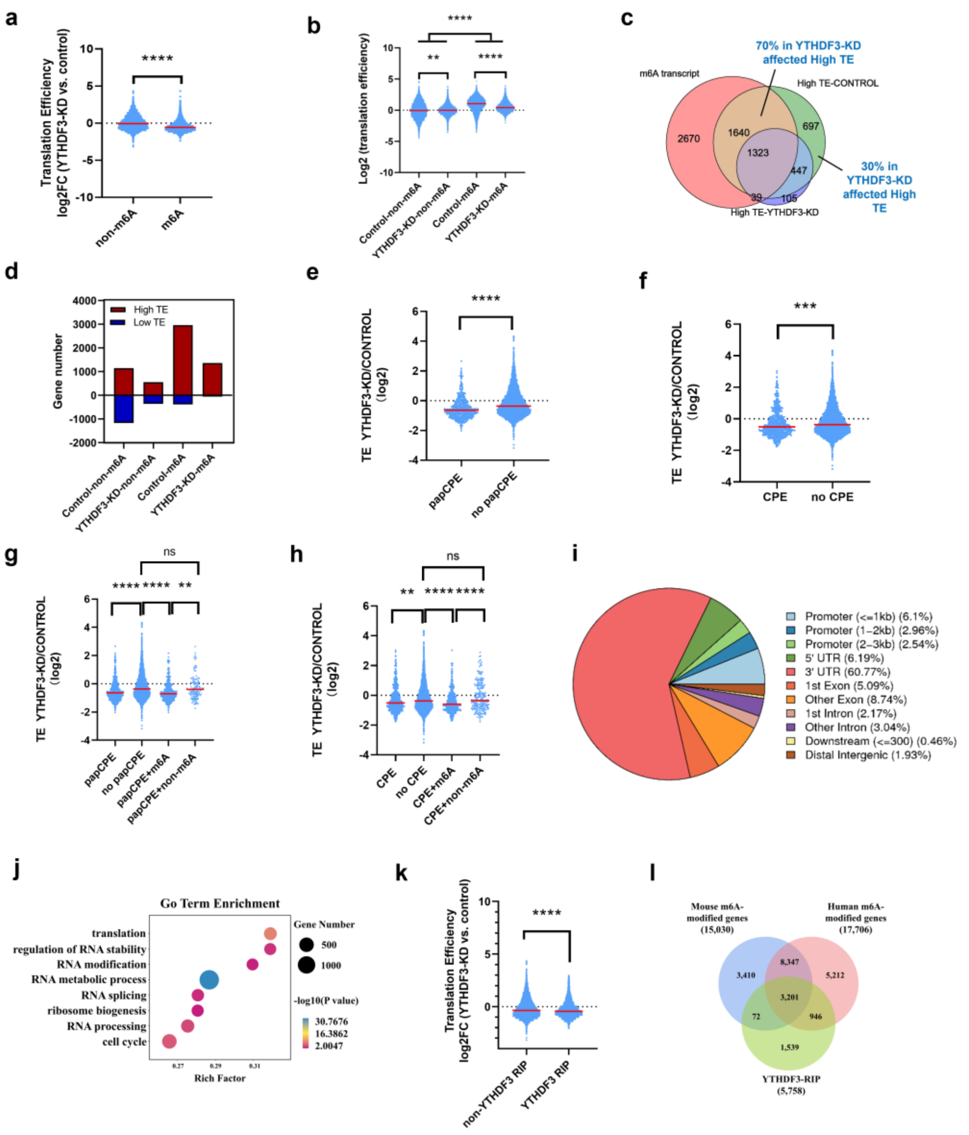

Figure S6. Translational efficiency analyses of oocytes upon YTHDF3 depletion. a)

Violin plots showing the TE changes in genes not enriched by m6A and m6A-enriched genes. p-Value was calculated with Student's t-test for independent samples. b) Violin plots showing the TE changes in group not enriched by m6A and m6A-enriched group genes in the YTHDF3-KD group oocytes compared with the control group oocytes. p-Values were calculated with one-way ANOVA and Bonferroni post-hoc test. c) Venn diagram showing the overlaps of the m6A-enriched gene set, high-TE genes in the control group oocytes, and high-TE genes in the YTHDF3-KD group oocytes. High TE, TE>2. d) Bar plots showing the numbers of high-TE genes (TE>2) and low-TE genes (TE<0.5) among the four groups of oocytes. Red denotes high-TE genes. Blue denotes low-TE genes. e) Violin plots showing the TE changes in genes of the papCPE-containing group and genes of the group not containing papCPE in the YTHDF3-KD group oocytes compared with the control group oocytes. p-Value was calculated with Student's t-test for independent samples. f) Violin plots showing the TE changes in the genes of the CPE-containing group and the genes of the group not containing CPE in the YTHDF3-KD group oocytes compared with the control group oocytes. p-Value was calculated with Student's t-test for independent samples. g) Violin diagram depicting the change in TE upon YTHDF3 depletion among the four groups of genes. p-Values were calculated with one-way ANOVA and Bonferroni post-hoc test. h) Violin plots showing the TE changes in four groups of genes in the YTHDF3-KD group oocytes and the control group oocytes. p-Values were calculated with one-way ANOVA and Bonferroni post-hoc test. i) The distribution and enrichment of YTHDF3-binding sites within different gene regions. j) GO term enrichment analysis of the overlapping YTHDF3 target genes described in Figure. 5c. k) Violin plots showing the TE changes in YTHDF3-target group and non-YTHDF3-target group genes in oocytes upon YTHDF3 depletion. p-Value was calculated with Student's t-test for independent samples. l) Overlapping analysis of human m6A-enriched genes (m6A-Atlas database), mouse m6A-containing genes (RMBase database), and YTHDF3 binding genes identified in HEK293T cells. YTHDF3-KD, YTHDF3 knockdown. TE, translational efficiency. CPEs, cytoplasmic polyadenylation elements. papCPE, CPEs residing near PAS, <100 nt. GO, Gene ontology. Ns, no significant difference. **$P<0.01$, ***$P<0.001$, ****$P<0.0001$.

Figure S7

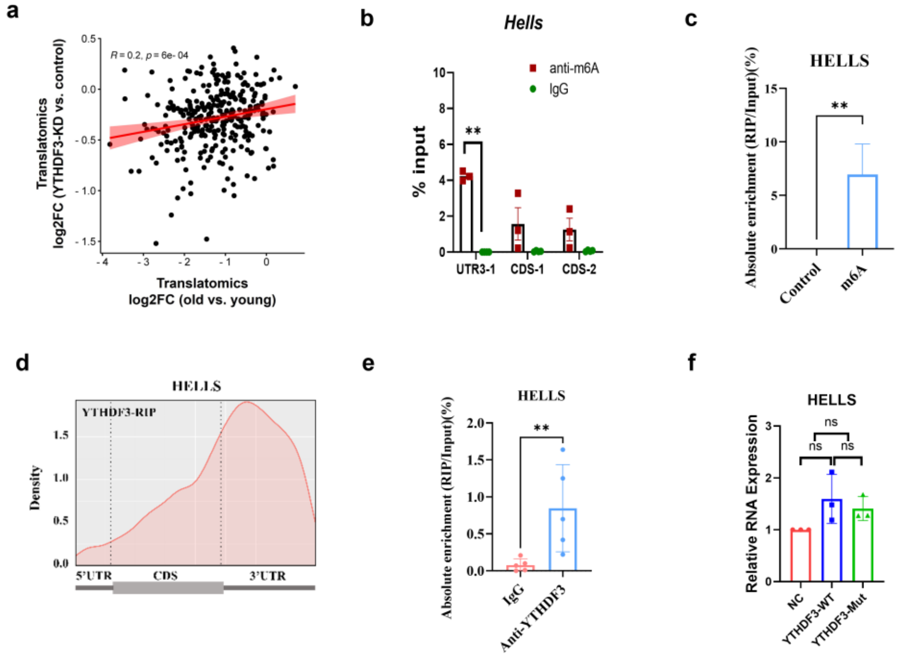

Figure S7. The RNA levels of *HELLS* genes identified by RT–qPCR in HEK293T cells. a) Scatter plot showing the correlation in gene translational log2 FC between YTHDF3-KD/control oocytes and aged/young oocytes. The *Pearson* correlation coefficient=0.2, with a *p*-Value < 0.001. b) Shown are m6A RIP followed by qPCR with 50 mouse oocytes on different sites of the *Hells* gene. UTR3-1, primers at the 3'UTR of *Hells*, CDS-1 and CDS-2, two pairs of primers at the CDS region of *Hells*. *p*-Value was calculated with two tailed Mann-Whitney test. c) M6A RIP followed by RT–qPCR in HEK293T cells confirming the enrichment of m6A in *HELLS* mRNA. *p*-Value was calculated with two tailed Mann-Whitney test. d) Distribution of the YTHDF3-binding site across the *HELLS* gene in HEK293T cells. e) YTHDF3 RIP followed by RT–qPCR in HEK293T cells confirming the interaction between *YTHDF3* and *HELLS* mRNA. *p*-Value was calculated with two tailed Mann-Whitney test. f) The RNA levels of the HELLS gene in HEK293T cells transfected with empty vector or wild-type (YTHDF3-WT) or mutant (YTHDF3-Mut) Flag-tagged YTHDF3 plasmid. *p*-Value was calculated with two tailed Mann-Whitney test. FC, fold change. RIP, RNA immunoprecipitation. NC, negative control. Ns, no significant difference. $^{**}P<0.01$.

Figure S8

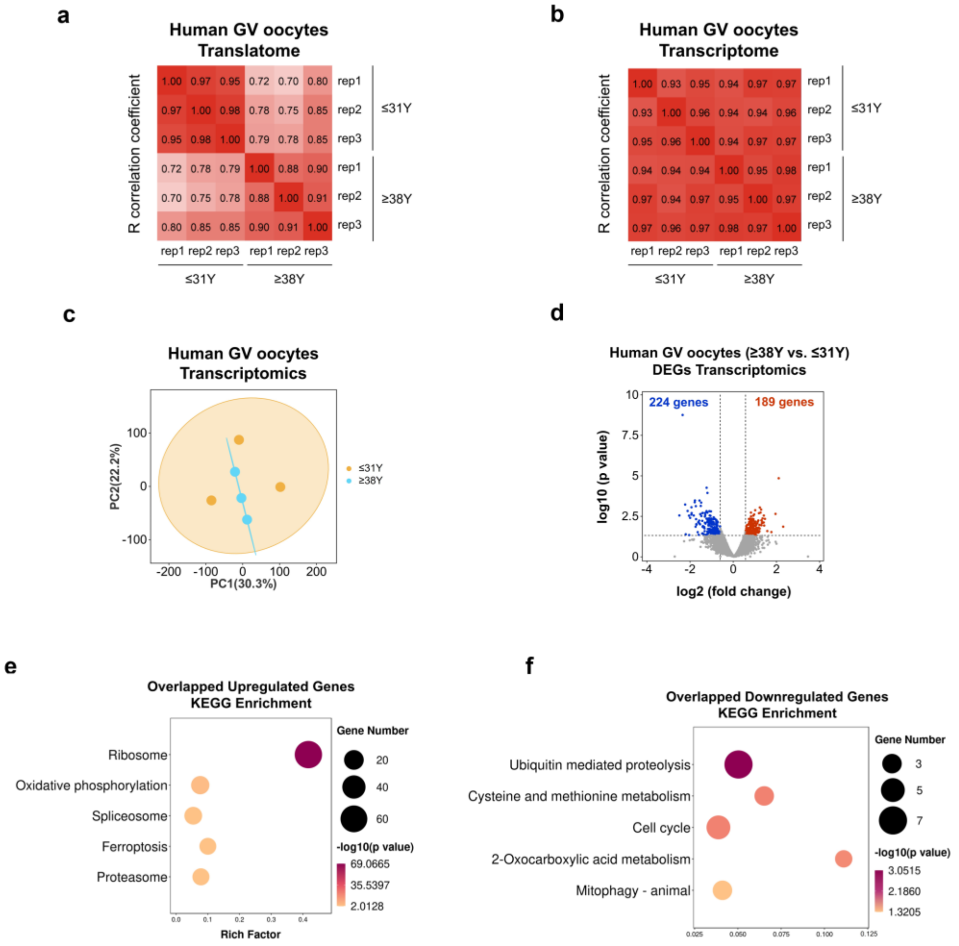

Figure S8. The transcriptional and translational landscapes of aged human oocytes. a) Heatmap depicting the *Pearson* correlation coefficient of the translatomics between each biological replicate from the young human oocytes and the aged human oocytes. b) Heatmap depicting the *Pearson* correlation coefficient of the transcriptomics between each biological replicate from the young human oocytes and the aged human oocytes. c) PCA plot of the transcriptomics of oocytes from young and aged human females. d) Volcano diagram showing DEGs detected by ultrasensitive transcriptomics. Red and blue dots denote up- and down-regulated genes, respectively. $P<0.05$, FC>1.5 or <0.67. e) Representative KEGG analysis of the overlapped translationally upregulated genes between aged mouse and human oocytes. f) Representative KEGG analysis of the overlapped translationally upregulated genes between aged mouse and human oocytes. PCA, principal component analysis. DEGs, differentially expressed genes. FC, fold change. KEGG, Kyoto Encyclopedia of Genes and Genomes.

Supplementary Tables:

Table S1. The changes of critical regulatory genes in transcriptomics during oocyte aging

Pathway	Gene ID	Gene Name	log2(FC)	*p*-Value
Oocyte meiosis	ENSMUSG00000017146	*Brca1*	-0.3826	0.375041652
	ENSMUSG00000041147	*Brca2*	-0.6041	0.107795584
	ENSMUSG00000022789	*Dnm1l*	0.0155	0.636930919
	ENSMUSG00000031928	*Mre11a*	-0.2108	0.853601771
	ENSMUSG00000026039	*Sgo2a*	-1.1184	0.010830307
	ENSMUSG00000094443	*Sgo2b*	-0.2814	0.723956467
RNA decay	ENSMUSG00000020362	*Cnot6*	-0.2726	0.714194381
	ENSMUSG00000034724	*Cnot6l*	0.0222	0.698922456
	ENSMUSG00000031601	*Cnot7*	0.7093	0.004437619
	ENSMUSG00000020515	*Cnot8*	0.0402	0.474688301
	ENSMUSG00000053110	*Yap1*	-1.1084	0.065332471
	ENSMUSG00000032056	*Btg4*	0.3878	0.052488237
	ENSMUSG00000035248	*Tut7*	0.0503	0.577225487
DNA methylation	ENSMUSG00000020661	*Dnmt3a*	-1.0151	0.036448999
	ENSMUSG00000027478	*Dnmt3b*	0.2871	0.144031217
	ENSMUSG00000004099	*Dnmt1*	-1.1192	1.81E-05
	ENSMUSG00000000730	*Dnmt3l*	-0.07	0.717579668
	ENSMUSG00000001228	*Uhrf1*	-0.5381	0.375945467
	ENSMUSG00000046323	*Dppa3*	0.3913	0.218123922
	ENSMUSG00000034832	*Tet3*	-1.0703	0.037639167
Histone modification	ENSMUSG00000006307	*Kmt2b*	-0.7119	0.134985059
	ENSMUSG00000036940	*Kdm1a*	-0.4466	0.390759249
	ENSMUSG00000038080	*Kdm1b*	-0.3985	0.395728995
	ENSMUSG00000029687	*Ezh2*	0.1552	0.177539376
	ENSMUSG00000017548	*Suz12*	-0.1309	0.968979422
	ENSMUSG00000030619	*Eed*	0.4956	0.020385722
	ENSMUSG00000044791	*Setd2*	-0.336	0.490615239
	ENSMUSG00000030801	*Kat8*	-0.0855	0.787175305
	ENSMUSG00000022521	*Crebbp*	0.0994	0.432672092
	ENSMUSG00000059288	*Cdyl*	-0.2509	0.736788661

Table S2. The changes of 302 identified genes in translational efficiency (TE) and translatomics

Gene Name	Old vs. Young			YTHDF3-KD vs. Control		
	TE (log2FC)	Translatomics (log2FC)	Translatomics *p*-Value	TE (log2FC)	Translatomics (log2FC)	Translatomics *p*-Value
Actr2	-3.2223	-2.3187	8.35332E-09	-1.2584	-1.2404	0.053927132
Amd1	-1.9912	-1.3155	0.000205853	-1.2019	-0.5871	0.144810839
Arid2	-3.7274	-3.1071	2.78063E-08	-2.0411	-0.809	0.387363087
Asap2	-0.6033	-0.9467	0.150304735	-0.9213	-0.8839	0.266157498
Capn7	-2.1271	-2.0312	5.5785E-09	-1.8646	-0.9809	0.296898379
Cdh2	-1.2928	-0.8637	0.007249401	-1.4264	-0.78	0.038984516
Ckap5	-1.9470	-2.1788	2.81287E-08	-1.1510	-0.5972	0.392521082
Clspn	-1.4549	-1.9972	0.001453223	-0.7024	-0.6199	0.414748233
Cul1	-1.3821	-1.0003	9.30016E-05	-1.2312	-0.6689	0.09285732
Ezh2	-1.3043	-0.809	0.020345546	-0.9651	-0.6054	0.100792202
Fbxo30	-2.0421	-1.0683	0.006140947	-1.4447	-0.735	0.095646965
Fem1b	-1.9542	-2.0309	1.35261E-06	-1.2032	-0.6877	0.364312174
Fignl1	-2.0246	-1.0681	0.015176017	-1.2452	-0.6215	0.158269249
Galnt1	-1.4930	-1.3633	0.03626276	-1.3156	-0.6682	0.210567329
Gdap1	-1.8142	-1.3303	0.002120321	-1.4716	-0.6061	0.157851108
Gsk3b	-2.9412	-2.7855	0.001974923	-1.3823	-0.6754	0.404300625
Hells	-1.8282	-1.6112	9.13826E-08	-0.7020	-0.6781	0.02672084
Ice1	-2.3970	-2.686	9.47681E-12	-1.0918	-0.7278	0.217306945
Ing5	-1.4842	-1.5113	0.007641374	-0.7816	-0.6122	0.412759773
Klhl9	-3.8141	-2.1653	3.08197E-08	-0.9623	-1.0723	0.132884284
Mki67	-3.0437	-3.2987	1.60976E-15	-1.5089	-0.809	0.293921071
Mllt10	-1.5226	-0.7878	0.002736263	-0.7394	-1.0499	0.071961435
Paip1	-2.4855	-1.4574	0.006948432	-2.0231	-1.4781	0.013773845
Pcdh9	-1.5185	-1.0034	0.010527822	-1.6062	-0.5905	0.296287419
Per3	-1.1065	-1.0322	0.006126983	-1.0778	-0.9963	0.122356309
Plk4	-1.7028	-1.1382	0.001620663	-1.2147	-0.7237	0.095229631
Pnpla8	-1.4793	-1.6031	4.5825E-07	-1.2334	-0.6288	0.399337697
Ptp4a2	-2.5962	-1.9637	2.00642E-08	-1.7110	-0.7945	0.088193569
Slc6a15	-1.9062	-0.8047	0.118569553	-1.6163	-0.939	0.054228047
Trip11	-2.0360	-2.4232	1.11941E-19	-0.9064	-0.6042	0.430452326
Tsc22d4	-0.6659	-1.4293	0.003467148	-0.6992	-0.7029	0.210669263
U2surp	-1.7222	-1.5208	0.010858743	-0.8268	-0.7722	0.201630246
Ubqln2	-1.6912	-1.8437	4.51443E-06	-1.8303	-1.0305	0.224128468
Zbtb18	-1.7766	-2.6925	2.51152E-05	-0.8190	-1.5209	0.088744519
Zbtb33	-1.7688	-2.1698	0.127266763	-1.1905	-0.7013	0.295117356
Zscan18	-1.0499	-1.8461	0.184237175	-1.0930	-0.9237	0.265535673

Table S3. RNA-binding proteins that were differently expressed between aged and young human oocytes

Gene ID	Gene Name	log2(fold change)	*p*-Value	FDR
ENSG00000124193	*SRSF6*	3.6524	5.91173E-07	2.67921E-05
ENSG00000169045	*HNRNPH1*	1.778	0.00358855	0.02237658
ENSG00000101811	*CSTF2*	-1.9793	6.54632E-06	0.000177679
ENSG00000149187	*CELF1*	-1.3724	0.00115512	0.009786672
ENSG00000122566	*HNRNPA2B1*	1.7183	0.005777245	0.031383861
ENSG00000164548	*TRA2A*	2.1947	0.001974808	0.014443136
ENSG00000116560	*SFPQ*	1.6989	0.000634935	0.00630015
ENSG00000169813	*HNRNPF*	-1.1269	0.00484292	0.027572268
ENSG00000197451	*HNRNPAB*	1.4801	0.009944283	0.045683675
ENSG00000204356	*NELFE*	1.9775	0.001136895	0.009699979
ENSG00000112081	*SRSF3*	1.4695	0.016672996	0.066849432
ENSG00000115875	*SRSF7*	1.8623	8.98992E-06	0.00022663
ENSG00000161547	*SRSF2*	2.2377	0.000644853	0.006351681
ENSG00000125870	*SNRPB2*	1.0583	0.014738892	0.061224841
ENSG00000197111	*PCBP2*	2.3066	4.55005E-05	0.000832319
ENSG00000168066	*SF1*	1.7769	0.011373385	0.050634157
ENSG00000132485	*ZRANB2*	1.659	0.001966858	0.014411117
ENSG00000003756	*RBM5*	1.9601	0.034477515	0.112104035
ENSG00000143889	*HNRNPLL*	3.001	6.03152E-06	0.000168361
ENSG00000147140	*NONO*	2.3205	3.09342E-05	0.000608649
ENSG00000119707	*RBM25*	1.1795	0.032355659	0.106972581
ENSG00000121057	*AKAP1*	-1.5879	0.003519094	0.022109448
ENSG00000126945	*HNRNPH2*	-1.4392	0.005735288	0.031240086
ENSG00000148840	*PPRC1*	-1.4868	0.000844589	0.007724829
ENSG00000265241	*RBM8A*	0.8896	0.043666424	0.131931476
ENSG00000004534	*RBM6*	1.5983	0.006068849	0.032529836
ENSG00000102317	*RBM3*	1.0402	0.042782245	0.129892498
ENSG00000151962	*RBM46*	-1.2209	0.027059635	0.093847838
ENSG00000099783	*HNRNPM*	1.4389	0.003897213	0.023817284
ENSG00000070756	*PABPC1*	1.6154	0.012794785	0.05535505
